# Genomic dissection of the correlation between milk yield and various health traits using functional and evolutionary information about imputed sequence variants of 34,497 German Holstein cows

**DOI:** 10.1186/s12864-024-10115-6

**Published:** 2024-03-09

**Authors:** Helen Schneider, Ana-Marija Krizanac, Clemens Falker-Gieske, Johannes Heise, Jens Tetens, Georg Thaller, Jörn Bennewitz

**Affiliations:** 1 Institute of Animal Science, University of Hohenheim, 70599 Stuttgart, Germany; 2 Department of Animal Sciences, University of Göttingen, 37077 Göttingen, Germany; 3 Vereinigte Informationssysteme Tierhaltung w.V. (VIT), 27283 Verden, Germany; 4 Institute of Animal Breeding and Husbandry, Christian-Albrechts University of Kiel, 24098 Kiel, Germany

**Keywords:** Dairy cattle, Health traits, Milk production, Genomic prediction, Genome annotation, Functional information, Imputed whole genome sequence variants

## Abstract

**Background:**

Over the last decades, it was subject of many studies to investigate the genomic connection of milk production and health traits in dairy cattle. Thereby, incorporating functional information in genomic analyses has been shown to improve the understanding of biological and molecular mechanisms shaping complex traits and the accuracies of genomic prediction, especially in small populations and across-breed settings. Still, little is known about the contribution of different functional and evolutionary genome partitioning subsets to milk production and dairy health. Thus, we performed a uni- and a bivariate analysis of milk yield (MY) and eight health traits using a set of ~34,497 German Holstein cows with 50K chip genotypes and ~17 million imputed sequence variants divided into 27 subsets depending on their functional and evolutionary annotation. In the bivariate analysis, eight trait-combinations were observed that contrasted MY with each health trait. Two genomic relationship matrices (GRM) were included, one consisting of the 50K chip variants and one consisting of each set of subset variants, to obtain subset heritabilities and genetic correlations. In addition, 50K chip heritabilities and genetic correlations were estimated applying merely the 50K GRM.

**Results:**

In general, 50K chip heritabilities were larger than the subset heritabilities. The largest heritabilities were found for MY, which was 0.4358 for the 50K and 0.2757 for the subset heritabilities. Whereas all 50K genetic correlations were negative, subset genetic correlations were both, positive and negative (ranging from -0.9324 between MY and mastitis to 0.6662 between MY and digital dermatitis). The subsets containing variants which were annotated as noncoding related, splice sites, untranslated regions, metabolic quantitative trait loci, and young variants ranked highest in terms of their contribution to the traits’ genetic variance. We were able to show that linkage disequilibrium between subset variants and adjacent variants did not cause these subsets’ high effect.

**Conclusion:**

Our results confirm the connection of milk production and health traits in dairy cattle via the animals’ metabolic state. In addition, they highlight the potential of including functional information in genomic analyses, which helps to dissect the extent and direction of the observed traits’ connection in more detail.

**Supplementary Information:**

The online version contains supplementary material available at 10.1186/s12864-024-10115-6.

## Background

Milk yields in dairy cattle have steadily risen during the last decades, which had been accelerated by genomic selection [1]. On the downside, claw diseases, infertility problems, and mastitis represent the most frequent reasons for culling [2] and it is undoubtedly that the cow’s health is negatively correlated to its production level [3]. This implies several issues for the dairy cattle industry, facing economical loss, ecological concerns, and a rising awareness about animal welfare in the professionals and the general society [4–6]. These concerns will become even more important with ongoing climate change and the continuously growing human population size. To reduce the environmental footprint of ruminants, researchers proposed several strategies like breeding for an improved herd efficiency and animal health or reduced greenhouse gas emissions [7–9]. Hereby, genomic prediction (GP) might play a major role, hence it is inevitable to continuously improve GP by dissecting the genetic basis of breeding traits [10].

At the moment, GP exploits mainly the long linkage disequilibrium (LD) blocks present in most livestock species. In GP, the effects of unknown quantitative trait loci (QTL) are estimated indirectly via genotypic markers that are in LD with the QTL, using a reference population [11]. For that reason, current multi-step GP is not robust to changes in the LD structure, making a continuous recalibration of marker effects indispensable [12]. Another limitation is that the accuracy of estimated genomic breeding values is below 1. Thereby, especially breeds that are genetically distant from the one where marker effects have been estimated experience a reduced accuracy [13–15].

To alleviate this problem at least partly, one can estimate QTL effects via markers in higher LD by increasing the marker density or by applying whole genome sequence (WGS) data, where causal variants are directly among the genotyped variants [15]. However, the latter is very cost intensive. As well, it has been shown that GP accuracies do not benefit from the application of WGS data (e.g., [16]). Thus, instead of merely increasing the marker density it is preferable to increase the amount of causal variants on the applied genotyping array [17].

However, even though many genome-wide association studies (GWAS) were performed with the aim to dissect the genetic architecture of complex traits, it is still challenging to identify causal variants [15, 18]. This is, because variants with a large and often deleterious effect are rather easy to detect, whereas variants with small effect sizes, typically found in complex traits due to their polygenic architecture, are not. In addition, by performing a GWAS one often yields a set of potential trait-associated variants being in high LD. Here, difficulties arise while selecting the causal variant among these potential variants [14], which would also require e.g., external validation sets. Although sequencing can be used to assess QTL more precisely [19], GWAS using sequence data still result in a set of trait-associated variants, which makes the final choice almost impossible [13, 20].

A vast majority of trait-associated variants are located in non-transcribed regions and most likely act functional, i.e., via changes in gene expression [13, 18, 20, 21]. Many studies highlight the importance of variants affecting transcription and translation for complex trait variation [21–24]. Recently, it had been shown that GP can be improved by applying functional information of variants either by using it as prior information for biological priors or by removing variants without functional importance [14, 25–28]. Thereby, it has been found that populations, which are already having high prediction accuracies using the common 50K chip, show only little or no advantage at all (e.g., [29]). Conversely, small breeds and across-breed settings, where prediction accuracies are usually low, benefit from including functional information in GP (e.g., [14, 26]). So far, Xiang et al. [20] analyzed 34 cattle traits, predominantly stature and milk production traits, using functional and evolutionary information of sequence variants. In detail, they used various sources of external information to define subsets based on the variants’ role in transcriptional and translational processes as well as their evolutionary background. Then, by estimating the variance each subset explained for the observed traits, they intended to detect subsets which would perform best in predicting causal mutations for complex cattle traits [20]. To our best knowledge, estimating variance components using a comparable amount of external information about sequence variants had neither been transferred to a bivariate setting nor to a set of various health traits. Thus, we aimed to study the contribution of 27 genome partitioning subsets, taken from Xiang et al. [20] to milk production and health traits as well as their genomic connection. We applied a set of 34,497 German Holstein cows, for which 50K genotypes, imputed WGS data containing ~ 17 million variants, and de-regressed proofs (DRP) of milk yield and eight health traits were available. The latter consisted of mastitis, four diseases belonging to the complex of claw diseases, and three diseases belonging to the complex of reproduction diseases.

The study is split into two parts. First, we performed a uni- and a bivariate variance component estimation within each subset. The bivariate analysis contrasted each health trait with milk yield, but the number of subsets was reduced to five to focus on the subsets, which contain sufficient genetic variance. In addition, we analyzed the subsets’ LD structure, MAF, and distribution over the genome. This latter step was meant to provide information whether a high effect of a subset is indeed because it contains causal variants or merely because of extensive LD, the subset variants’ MAF or their distribution over the genome. We expect to identify subsets with a significant contribution to the heritability and genetic correlation between milk production and health traits. This knowledge might help to enhance the understanding of biological mechanisms linking these traits.

## Methods

### Material

The phenotypic data was provided by the national computing center (Vereinigte Informationssysteme Tierhaltung w.V., Verden, Germany). We analyzed 34,497 German Holstein cows with DRPs for milk yield (MY) and eight health traits, whose first lactation was between 2015 and 2020. A detailed description of the filtering can be taken from Schneider et al. [30]. The DRPs were based on on-farm recordings of disease cases, recorded by the farmer as well as veterinarians and claw trimmers. We analyzed the following claw diseases: claw ulcers (CU), digital dermatitis (DD), interdigital hyperplasia (IH), and digital phlegmon (PH). Additionally, mastitis (MAS) and the three reproduction diseases metritis (MET), retained placenta (RP), and cyclus disturbances (CD) were examined. Table 1 provides an overview over the amount of individuals that were available for the analysis of each trait. To avoid confusion, it has to be noted that the DRP for the health traits were transformed such that a higher value is favorable in terms of animal health.

**Table 1:** Number of individuals with deregressed proofs and 50K chip heritabilities and genetic correlations

Trait	Trait abbreviation	Individuals No.	${h}^{2}$(se)	${r}_{g}$(se)
Milk yield	MY	34,497	0.4358 (0.0078)	X
Interdigital hyperplasia	IH	30,968	0.1530 (0.0069)	-0.1059 (0.0271)
Digital phlegmon	PH	26,437	0.0980 (0.0062)	-0.1816 (0.0319)
Claw ulcers	CU	27,012	0.1530 (0.0072)	-0.0685 (0.0280)
Digital dermatitis	DD	30,056	0.1747 (0.0072)	-0.0187 (0.0262)
Mastitis	MAS	33,298	0.1326 (0.0063)	-0.3030 (0.0261)
Metritis	MET	27,283	0.0558 (0.0048)	-0.0111 (0.0387)
Retained placenta	RP	28,182	0.0738 (0.0053)	-0.0878 (0.0349)
Cyclus disturbances	CD	26,884	0.0771 (0.0055)	-0.1970 (0.0341)

Shown are the heritabilities (${{\text{h}}}^{2}$, from model M1), genetic correlations (${{\text{r}}}_{{\text{g}}}$, from model M2) and the corresponding standard errors (se). Genetic correlations were estimated between each health trait and milk yield

### Genotypes

50 K chip genotypes, provided by the vit, and imputed WGS data was available for our analyses. The imputation is described in Krizanac et al. [31]. For the 50K chip, 44,126 variants remained after filtering out variants on sex chromosomes and those with a minor allele frequency (MAF) below 0.01. We applied the same filter steps but increased the MAF threshold to 0.05 for the imputed WGS variants. Additionally, the quality of the imputed WGS dataset was assessed using the dosage R-squared parameter (DR2). The DR2 parameter serves as a quality control of imputed datasets since it estimates the squared correlation between the estimated and true allele dosage [32]. Variants with a DR2 < 0.75 were removed [31]. Finally, a total of 16,882,734 variants were left for the analysis. The imputed WGS dataset was divided into 27 subsets of genome partitioning categories, which were defined following the approach from Xiang et al. [20]. Below, we will briefly describe the definition and detection of each category. The number of variants per subset can be taken from Table 2. First, 11 subsets were defined using the output of the LD score calculation with the GCTA software version 1.92.3 beta3 [33]. We set the window size to 50 kbp and received the LD score of each variant. As a byproduct, the output also provides the MAF and the number of variants within the window of 50 kbp (variant density, VD) for each variant in a separate column (“snp_num”). Using these three columns, we split the variants into quartiles to define the LD, VD, and MAF quartiles, where the lowest quartile (e.g., LD1) has the lowest LD, VD or MAF. Since the variance explained by the MAF1 quartile was only very minor in the study from Xiang et al. [20], we decided not to include this subset in our analysis.

**Table 2:** *Across trait per variant* *h*^2^ from model M3 and number of variants for each subset

Subset	*across-trait per variant h*^2^	Variants No.
50K	$3.409*{10}^{-6}$	44,126
splice sites	$2.766*{10}^{-6}$	7,308
mQTL	$2.304*{10}^{-6}$	5,179
untranslated regions	$1.206*{10}^{-6}$	28,039
noncoding related	$8.537*{10}^{-7}$	3,189
young	$9.663*{10}^{-8}$	88,195
selection signatures	$6.171*{10}^{-8}$	1,138
VD1	$1.930*{10}^{-8}$	4,205,241
LD2	$1.732*{10}^{-8}$	4,220,653
LD1	$1.589*{10}^{-8}$	4,220,680
VD2	$9.576*{10}^{-9}$	4,225,644
MAF3	$8.440*{10}^{-9}$	4,220,866
LD3	$7.779*{10}^{-9}$	4,220,703
coding related	$7.303*{10}^{-9}$	68,787
MAF2	$6.260*{10}^{-9}$	4,220,702
MAF4	$5.584*{10}^{-9}$	4,220,595
VD3	$4.606*{10}^{-9}$	4,211,960
geQTL	$4.552*{10}^{-9}$	87,955
intergenic	$2.347*{10}^{-9}$	8,037,337
VD4	$2.134*{10}^{-9}$	4,237,933
Conserved 100	$1.984*{10}^{-9}$	240,145
LD4	$1.551*{10}^{-9}$	4,220,696
gene end	$5.099*{10}^{-10}$	676,873
ChIPseq	$4.121*{10}^{-10}$	783,523
sQTL	$3.068*{10}^{-10}$	907,930
eeQTL	$2.268*{10}^{-10}$	787,213
aseQTL	$2.027*{10}^{-10}$	826,089
intron	$1.666*{10}^{-10}$	3,071,141

Next, seven subsets were defined based on their category of functional annotation, taken from Ensembl variant effect predictor [34] and NGS variant [35]. In detail, those were the subsets comprising noncoding-related variants (noncoding related), coding-related variants (coding related), intergenic and gene end variants (gene end), as well as variants located in untranslated regions (UTR), splice sites, and introns. Some annotation categories had to be merged in order to achieve subsets with a sufficient number of variants. As an example, this means that variants annotated as “noncoding_transcript_exon_variant”, “noncoding_transcript_variant”, and “mature_miRNA_variant” were merged to the noncoding related subset [20].

Another nine subsets were based on preliminary discovery analyses. We thankfully received the information about which variants belong to these subsets from Xiang et al. [20]. Further details about these subsets and their definition can be taken from their publication. The subsets’ definition and discovery is briefly explained in the following. Five subsets fall into the category of intermediate QTL, namely the gene expression QTL (geQTL), exon expression QTL (eeQTL), splicing QTL (sQTL), allele specific expression QTL (aseQTL), and polar lipid metabolite QTL (mQTL). The geQTL, eeQTL, and sQTL were detected in a previous study [24] and further processed in a meta-analysis [20]. Variants falling into the category of aseQTL were found using RNAseq data from Bouwman et al. [23] and the methodology of Khansefid et al. [36]. The discovery of mQTL applied metabolite data extracted by Liu et al. [37]. Next, variants were chosen that were located under ChIPseq peaks in previous studies on bovine muscle and liver tissue [38, 39]. Together with variants found in a ChIPseq analysis of bovine tissue from the mammary gland that was performed by Xiang et al. [20], they were merged into the ChIPseq subset, which reflects variants affecting DNA-protein interactions.

When it comes to the evolutionary history of variants, three sources of external information were chosen to split the variants into different categories. Firstly, it had been demonstrated in humans that, compared to neutral selection, recent selection evokes an increased frequency of favorable alleles [40]. Thus, Xiang et al. [20] assumed that variants, which have been under recent selection, are enriched in regions where the positive correlation with rare variants is low. Following this assumption, they defined a subset of young variants based on their positive correlation with rare variants. Next, variants annotated as selection signatures were defined as the ones showing a significant (*p* <0.0001) association with the cattles’ beef or dairy phenotype [20]. The last subset (conserved across 100 species, CONS100) consisted of variants that showed a high degree of phylogenetic conservation across 100 species according their PhastCons Score [41]. The PhastCons Score was calculated across these 100 species [20].

### Statistical analysis

First, we performed a univariate variance component estimation for each trait with the following mixed linear model (M1) using GCTA software version 1.92.3 beta3 [33].

1 $$\varvec{y}=\upmu1+\varvec{Z}_{50\varvec{K}}\varvec{g}_{50\varvec{K}}+\varvec{e}$$

Here, the vector ${\varvec{y}}$ contains the DRP of each animal, $\upmu$ denotes the mean, and ${1}$ is a vector of 1s. Vector ${\varvec{e}}$ is the residual and vector ${{\varvec{g}}}_{50{\varvec{K}}}$ the polygenic term with ${{\varvec{Z}}}_{50{\varvec{K}}}$ as the design matrix. It was assumed that both terms follow a normal distribution with ${{\varvec{g}}}_{50{\varvec{K}}} \sim N(0, {{\varvec{G}}}_{50{\varvec{K}}}{\sigma }_{g,50K}^{2})$ and ${\varvec{e}} \sim N(0, {\varvec{I}}{\sigma }_{{\varvec{e}}}^{2})$, whereby ${\sigma }_{g,50K}^{2}$ is the additive genetic and ${\sigma }_{e}^{2}$ the residual variance. ${\varvec{I}}$ denotes the identity matrix and ${{\varvec{G}}}_{50{\varvec{K}}}$ the additive genetic relationship matrix (GRM) of the 50K chip, which was computed using GCTA [42]. While constructing the GRM, all 34,497 animals were used.

Then, we applied model **M2**

2 $$\left[\begin{array}{c}{{\varvec{y}}}_{1}\\ {{\varvec{y}}}_{2}\end{array}\right] =\left[\begin{array}{c}{\upmu }_{1}{1}\\ {\upmu }_{2}{1}\end{array}\right]+\left[\begin{array}{cc}{{\varvec{Z}}}_{50{\varvec{K}},1}& 0\\ 0& {{\varvec{Z}}}_{50{\varvec{K}},2}\end{array}\right] \left[\begin{array}{c}{{\varvec{g}}}_{50{\varvec{K}},1}\\ {{\varvec{g}}}_{50{\varvec{K}},2}\end{array}\right]+\left[\begin{array}{c}{{\varvec{e}}}_{1}\\ {{\varvec{e}}}_{2}\end{array}\right]$$

to estimate variance components for eight trait-combinations, each contrasting MY with one of the eight health traits. ${{\varvec{y}}}_{1}$ and ${{\varvec{y}}}_{2}$ are the vectors containing the DRPs of trait 1 (MY) and trait 2 (one of the eight health traits) with their means ${\upmu }_{1}$ and ${\upmu }_{2}$. Vectors ${{\varvec{g}}}_{50{\varvec{K}},1}$ (${{\varvec{g}}}_{50{\varvec{K}},2}$) and ${{\varvec{e}}}_{1}$ (${{\varvec{e}}}_{2}$) are the corresponding polygenic and residual terms. ${{\varvec{Z}}}_{50{\varvec{K}},1}$ and ${{\varvec{Z}}}_{50{\varvec{K}},2}$ denote the design matrices. The variance-covariance-matrix was modeled as

3 $$var\left[\begin{array}{c}{{\varvec{g}}}_{50{\varvec{K}},1}\\ {{\varvec{g}}}_{50{\varvec{K}},2}\\ \begin{array}{c}{{\varvec{e}}}_{1}\\ {{\varvec{e}}}_{2}\end{array}\end{array}\right]=\left[\begin{array}{cc}{{\varvec{G}}}_{50{\varvec{K}}}{\sigma }_{g,50K,1}^{2}& {{{\varvec{G}}}_{50{\varvec{K}}}\sigma }_{g,50K,12}\\ {{\varvec{G}}}_{50{\varvec{K}}}{\sigma }_{g,50K,21}& {{\varvec{G}}}_{50{\varvec{K}}}{\sigma }_{g,50K,2}^{2}\\ \begin{array}{c}0\\ 0\end{array}& \begin{array}{c}0\\ 0\end{array}\end{array} \begin{array}{cc}0& 0\\ 0& 0\\ \begin{array}{c}{\varvec{I}}{\sigma }_{e,1}^{2}\\ {{\varvec{I}}\sigma }_{e21}\end{array}& \begin{array}{c}{{\varvec{I}}\sigma }_{e,12}\\ {\varvec{I}}{\sigma }_{e,2}^{2}\end{array}\end{array}\right]$$

Here, ${\sigma }_{g,50K,1}^{2}$ and ${\sigma }_{g,50K,2}^{2}$ (${\sigma }_{e,1}^{2}$ and ${\sigma }_{e,2}^{2}$) are the additive genetic (residual) variance and ${\sigma }_{g,50K,12}$ and ${\sigma }_{g,50K,21}$ (${\sigma }_{e,12}$ and ${\sigma }_{e,21}$) the respective covariance. Heritabilities (${h}^{2}$) and genetic correlations (${r}_{g}$) were calculated using standard notations.

Afterwards, our aim was to estimate variance components for each subset. Thus, we conducted a set of uni- and bivariate analyses of the same traits and trait-combinations as with models M1 and M2 but included two polygenic terms, one for the respective subset and one for the 50K chip. We applied both terms following the approach of Xiang et al. [20]. The underlying idea is that every large set of variants might explain a lot of genetic (co-)variance if these variants are in high LD with surrounding variants. Therefore, by applying two polygenic terms, we seek for a set of sequence variants explaining additional (co-)variance to the one explained by the common variants on the 50K chip. If a set explains additional (co-)variance, we expect that this points to a set containing a potential causal mutation. Here, either the causal mutation itself is among the subset variants or they are in higher LD with it than the variants on the 50K chip.

In the univariate analysis, the following model (M3)

4 $${\varvec{y}}=\upmu{1}+{{\varvec{Z}}_{\varvec{i}}{\varvec{g}}}_{{\varvec{i}}}+{{{\varvec{Z}}}_{50{\varvec{K}}}{\varvec{g}}}_{50{\varvec{K}}}+{{\varvec{e}}}_{{\varvec{i}}}$$

was applied. Here, vector ${{\varvec{g}}}_{{\varvec{i}}}$ represents the polygenic and vector ${{\varvec{e}}}_{{\varvec{i}}}$ the residual term of the $i$-th subset, whereby ${{\varvec{Z}}}_{{\varvec{i}}}$ is the design matrix. Vector ${{\varvec{g}}}_{50{\varvec{K}}}$ denotes the corresponding polygenic term of the 50K chip. Again, we assumed that they follow a normal distribution with ${{\varvec{e}}}_{{\varvec{i}}} \sim N\left(0, {\varvec{I}}{\sigma }_{e,i}^{2}\right)$, ${{\varvec{g}}}_{{\varvec{i}}} \sim N(0, {{\varvec{G}}}_{{\varvec{i}}}{\sigma }_{{g,}_{i}}^{2})$, and ${{\varvec{g}}}_{50{\varvec{K}}} \sim N(0, {{\varvec{G}}}_{50{\varvec{K}}}{\sigma }_{g,50K}^{2})$. The GRM of the $i$-th subset, ${{\varvec{G}}}_{{\varvec{i}}}$, was computed using GCTA. Heritabilities for the $i$-th subset were calculated with the as

5 $${h}_{set,i}^{2}= \frac{{\sigma }_{g,i}^{2}}{{\sigma }_{g,50K}^{2}+ {\sigma }_{g,i}^{2}+{\sigma }_{e,i}^{2}},$$

and the corresponding heritabilities for the 50K chip while analyzing the $i$-th subset as

6 $${h}_{50K}^{2}= \frac{{\sigma }_{g,50K}^{2}}{{\sigma }_{g,50K}^{2}+ {\sigma }_{g,i}^{2}+{\sigma }_{e,i}^{2}}.$$

We performed the univariate analysis for each trait (9) within each subset (27), yielding 243 estimates for ${h}_{set}^{2}$. In order to differentiate between subsets that have a high effect because they contain a lot of variants, and those with a high effect because they contain causal variants, we computed the *across trait per variant* ${h}^{2}$ of each subset. To do so, the sum of the ${h}_{set}^{2}$ estimates for each trait within the respective subset was divided by the number of traits (9) and the number of variants within this subset. Additionally, this was done for each trait at a time, resulting in the *trait-specific per variant* ${h}^{2}$. This calculation divided the ${h}_{set}^{2}$ estimates by the number of variants within the respective subset. Both parameters were also calculated for the heritability estimates from model M1.

In the bivariate setting, we reduced the number of subsets to five by choosing those having the highest *across trait per variant* ${h}^{2}$ (UTR, noncoding related, splice sites, mQTL, and young). This was done to focus on the subsets with a noteworthy amount of genetic variance. Variance components were here estimated with the following mixed linear model (M4).

7 $$\left[\begin{array}{c}{{\varvec{y}}}_{1}\\ {{\varvec{y}}}_{2}\end{array}\right] =\left[\begin{array}{c}{\upmu }_{1}{1}\\ {\upmu }_{2}{1}\end{array}\right]+\left[\begin{array}{cc}{{\varvec{Z}}}_{{\varvec{i}},1}& 0\\ 0& {{\varvec{Z}}}_{{\varvec{i}},2}\end{array}\right]\left[\begin{array}{c}{{\varvec{g}}}_{{\varvec{i}},1}\\ {{\varvec{g}}}_{{\varvec{i}},2}\end{array}\right]+\left[\begin{array}{cc}{{\varvec{Z}}}_{50{\varvec{K}},1}& 0\\ 0& {{\varvec{Z}}}_{50{\varvec{K}},2}\end{array}\right] \left[\begin{array}{c}{{\varvec{g}}}_{50{\varvec{K}},1}\\ {{\varvec{g}}}_{50{\varvec{K}},2}\end{array}\right]+\left[\begin{array}{c}{{\varvec{e}}}_{{\varvec{i}},1}\\ {{\varvec{e}}}_{{\varvec{i}},2}\end{array}\right]$$

For traits 1 (MY) and 2 (each of the eight health traits), ${{\varvec{g}}}_{{\varvec{i}},1}$ (${{\varvec{g}}}_{{\varvec{i}},2})$ and ${{\varvec{g}}}_{50{\varvec{K}},1}$ (${{\varvec{g}}}_{50{\varvec{K}},2}$) denote the polygenic terms for the $i$-th subset and the 50K chip, respectively, with ${{\varvec{Z}}}_{{\varvec{i}},1}$ (${{\varvec{Z}}}_{{\varvec{i}},2}$) and ${{\varvec{Z}}}_{50{\varvec{K}},1}$ (${{\varvec{Z}}}_{50{\varvec{K}},2}$) as the corresponding incidence matrices. ${{\varvec{e}}}_{{\varvec{i}},1}$ (${{\varvec{e}}}_{{\varvec{i}},2}$) is the respective residual term. The variance-covariance structure between these three terms was

8 $$var\left[\begin{array}{c}{{\varvec{g}}}_{50{\varvec{K}},1}\\ {{\varvec{g}}}_{50{\varvec{K}},2}\\ \begin{array}{c}{{\varvec{g}}}_{{\varvec{i}},1}\\ {{\varvec{g}}}_{{\varvec{i}},2}\\ \begin{array}{c}{{\varvec{e}}}_{{\varvec{i}},1}\\ {{\varvec{e}}}_{{\varvec{i}},2}\end{array}\end{array}\end{array}\right]=\left[\begin{array}{ccc}\begin{array}{cc}{{\varvec{G}}}_{50{\varvec{K}}}{\sigma }_{g,50K,1}^{2}& {{{\varvec{G}}}_{50{\varvec{K}}}\sigma }_{g,50K,12}\\ \begin{array}{c}{{{\varvec{G}}}_{50{\varvec{K}}}\sigma }_{g,50K,21}\\ 0\\ \begin{array}{c}0\\ 0\\ 0\end{array}\end{array}& \begin{array}{c}{{\varvec{G}}}_{50{\varvec{K}}}{\sigma }_{g,50K,2}^{2}\\ 0\\ \begin{array}{c}0\\ 0\\ 0\end{array}\end{array}\end{array}& \begin{array}{c}0\\ 0\\ \begin{array}{c}{{\varvec{G}}}_{{\varvec{i}}}{\sigma }_{g,i,1}^{2}\\ {{\varvec{G}}}_{{\varvec{i}}}{\sigma }_{g,i,21}\\ \begin{array}{c}0\\ 0\end{array}\end{array}\end{array}& \begin{array}{ccc}\begin{array}{c}0\\ 0\\ \begin{array}{c}{{{\varvec{G}}}_{{\varvec{i}}}\sigma }_{g,i,12}\\ {{\varvec{G}}}_{{\varvec{i}}}{\sigma }_{g,i,2}^{2}\\ \begin{array}{c}0\\ 0\end{array}\end{array}\end{array}& \begin{array}{c}0\\ 0\\ \begin{array}{c}0\\ 0\\ \begin{array}{c}{\varvec{I}}{\sigma }_{e,1}^{2}\\ {{\varvec{I}}\sigma }_{e21}\end{array}\end{array}\end{array}& \begin{array}{c}0\\ 0\\ \begin{array}{c}0\\ 0\\ \begin{array}{c}{{\varvec{I}}\sigma }_{e,12}\\ {\varvec{I}}{\sigma }_{e,2}^{2}\end{array}\end{array}\end{array}\end{array}\end{array}\right].$$

${\sigma }_{g,50K,1}^{2}$ and ${\sigma }_{g,50K,2}^{2}$ (${\sigma }_{g,i,1}^{2}$ and ${\sigma }_{g,i,2}^{2}$) contain the additive genetic variance of traits 1 and 2, explained by the 50K chip ($i$-th subset). ${\sigma }_{g,50K, 12}$ (${\sigma }_{g,i, 12})$ and ${\sigma }_{g,50K, 21}$ (${\sigma }_{g,i, 21})$ denote the genetic covariance and ${\sigma }_{e,1}^{2}$ (${\sigma }_{e,2}^{2})$ and ${\sigma }_{e, 12}$ (${\sigma }_{e, 21}$) the residual variance (covariance). Hereinafter, we refer to the subset genetic correlations with the term ${r}_{g,set}$, which is calculated as

9 $${r}_{g,set,i}= \frac{{\sigma }_{g,i, 12}}{\sqrt{{\sigma }_{g,i,1}^{2}* {\sigma }_{g,i,2}^{2}}}$$

for the $i$-th subset. Corresponding genetic correlations for the 50K chip, ${r}_{g,50K}$ were computed with a similar formula that contained the (co-)variance terms of the 50K chip.

In line with the univariate analysis, we obtained the *trait-specific per variant* ${r}_{g}$ by dividing each ${r}_{g,set}$ as well as each ${r}_{g}$ estimate by the number of variants within the respective subset for the ${r}_{g,set}$ estimates or the 50K chip for the ${r}_{g}$ estimates. We did not calculate the *across trait per variant* ${r}_{g}$ since genetic correlations can be both, positive and negative, and summing them up as it is done for the *across trait per variant* ${h}^{2}$ is not straightforward.

Since genetic correlations do not provide information about the contribution of each covariance term to the total covariance, we defined three additional parameters to obtain information about this extent. Those were the ${relcov}_{set}$, the ${relcov}_{50K}$, and the ${relcov}_{e}$. For the $i$-th subset, ${relcov}_{set}$ was calculated as

10 $${relcov}_{set,i}=\frac{\sigma_{g,i,12}}{\left|\sigma_{g,50K,12}\right|+\left|\sigma_{g,i,12}\right|+\left|\sigma_{e,i,12}\right|},$$

$${relcov}_{50K}$$ as

11 $${relcov}_{50K,i}=\frac{\sigma_{g,50K,12}}{\left|\sigma_{g,50K,12}\right|+\left|\sigma_{g,i,12}\right|+\left|\sigma_{e,i,12}\right|},$$

and $${relcov}_{e}$$ as

12 $${relcov}_{e,i}=\frac{\sigma_{e,i,12}}{\left|\sigma_{g,50K,12}\right|+\left|\sigma_{g,i,12}\right|+\left|\sigma_{e,i,12}\right|}.$$

### LD analysis

It might occur that some subsets explain more genetic variance because of extensive LD in the genome rather than harboring causal variants. As mentioned above, the 50K GRM was incorporated in models M3 and M4 to account for the variance of common variants in high LD. However, differences in the MAF properties of 50K and sequence variants might evoke that this procedure did not account for all LD biased variance of the partitionings. Therefore, we examined the LD structure, MAF, and distribution over the genome of each subset. Six parameters were defined and will be explained in the following. Concerning the LD structure, we differentiate between the LD of subset variants with other surrounding subset variants (subset-intern) and surrounding sequence variants that do not belong to the respective subset (subset-extern). Then, we calculated the Pearson correlations between each of these parameters and the *across trait per variant* ${h}^{2}$ using the *cor* function of R version 4.0.4 [43].

For every subset, we calculated the LD of each subset variant with every other sequence variant within a window of 500 kilobasepairs (kbp) using PLINK version 1.9 [44]. Within this window, sequence variants are either also part of this subset (subset variants) or not part of this subset (adjacent sequence variants). The output of the LD calculation reports inter-variant correlations for all subset variants with both.

For the first and second parameter, the output was filtered in the way that we removed inter-variant correlations between each subset variant and other subset variants. Then, the first parameter, *mean ld extern*, was calculated as the mean ${r}^{2}$ of the remaining variant pairings. The corresponding decay of LD (*decay extern*) was defined as the proportion of the mean ${r}^{2}$ between 120 and 500 kbp to the mean ${r}^{2}$ up to a distance of 25 kbp. A lower value of *decay extern* indicates a rapid decay whereas a higher value points to a slow one. The third and fourth parameter, *mean ld intern* and *decay intern,* were obtained in the same way as the *mean ld extern* and *decay extern.* The difference was that we removed inter-variant correlations between each subset variant and adjacent sequence variants from the output. For all four parameters, a positive correlation with the *across trait per variant* ${h}^{2}$ indicates that the subset’s effect is increasing with rising LD between the subset variants and between subset variants and adjacent sequence variants.

Next, the parameter *distribution* was defined as the proportion of variant pairs that remained after removing all variant pairs between subset variants and adjacent sequence variants from the output to the number of variants pairs in the unfiltered output. This parameter aims to provide information about the distribution of the subset variants over the genome. A lower value means that more variant pairs were removed, which indicates that this subset’s variants appear more accumulated than in subsets with a higher value. If the correlation with the *across trait per variant* ${h}^{2}$ is positive, we assume that subsets, whose variants’ distribution over the genome is more equal, explain more genetic variance. The last parameter was the mean MAF of the subset variants (*mean MAF)*, which was a by-product of the LD calculation. Here, a positive correlation with the *across trait per variant* ${h}^{2}$ indicates that subsets with a higher MAF explain more genetic variance.

## Results

### Variance component estimation

Heritabilities from model M1 were low to moderate for the health traits and high for MY, ranging from 0.0558 for MET to 0.4358 for MY (Table 1). In contrast, the minimum ${h}_{set}^{2}$ was very low with <0.0001 for some traits and subsets. The highest ${h}_{set}^{2}$ estimates were for MY in the subsets VD1 (0.2757), MAF3 (0.1358), LD2 (0.1216), and UTR (0.1094) (Table S1). Concerning the health traits, we found moderate subset heritabilities in the LD1 subset for CU (0.1044) and IH (0.1211) (Table S1). We observed that the subsets containing fewer variants had a slightly higher *across trait per variant* ${h}^{2}$. Next to the 50K chip with an *across trait per variant* ${h}^{2}$ of $3.409*{10}^{-6}$, the splice sites subset ranked highest with a value of $2.766*{10}^{-6}$, followed by mQTL, UTR, noncoding related, and young variants. Least *across trait per variant* ${h}^{2}$ was explained by the intron subset ($1.666*{10}^{-10}$) (Table 2).

**Fig. 1:**
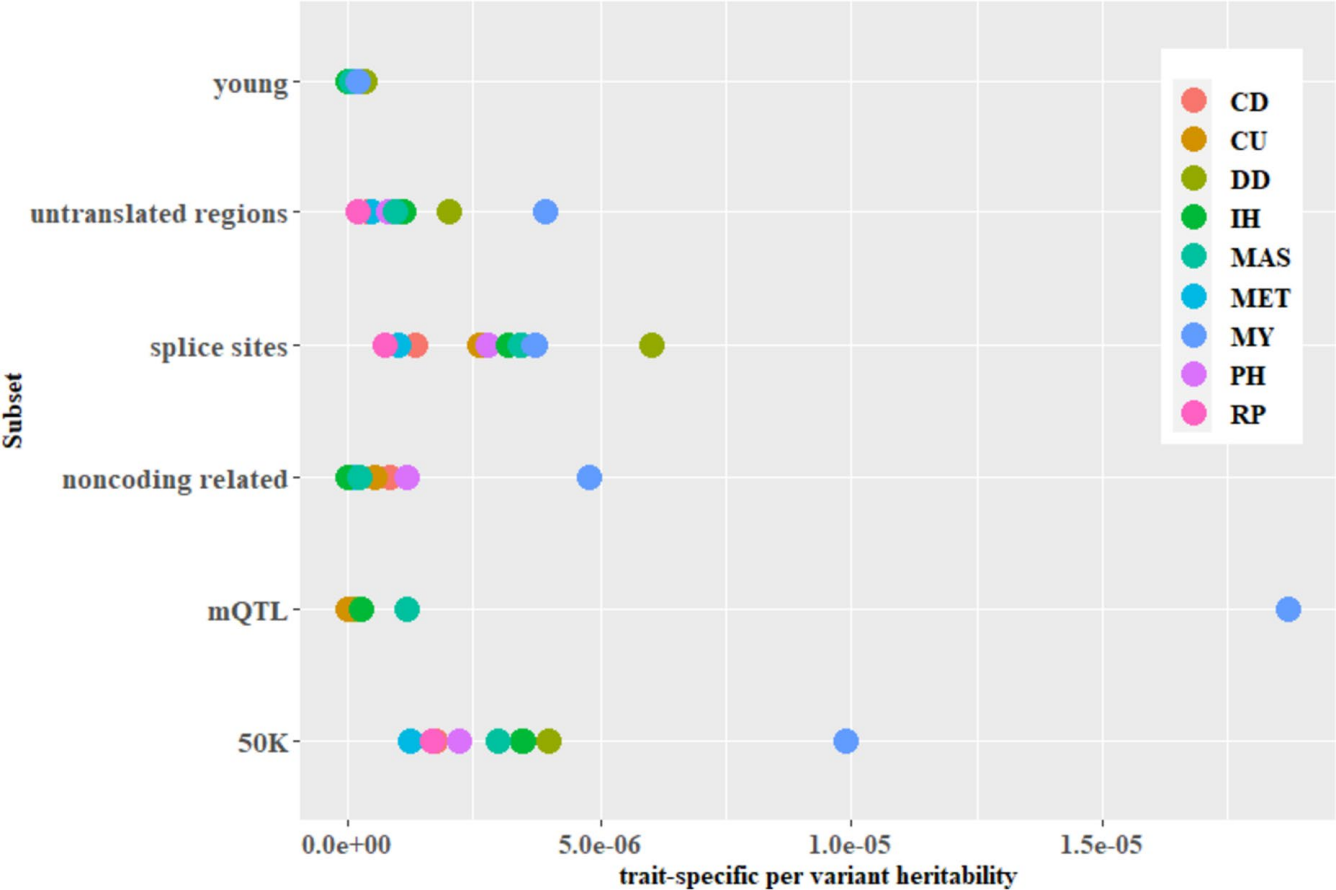
*Trait-specific per variant* ${h}^{2}$ of the subsets applied to the bivariate analysis and the 50K chip. The traits are cyclus disturbances (CD), retained placenta (RP), metritis (MET), mastitis (MAS), digital dermatitis (DD), interdigital hyperplasia (IH), digital phlegmon (PH), claw ulcers (CU), and milk yield (MY). Results from model M3

For MY, the *trait-specific per variant* ${h}^{2}$ was highest for the 50K chip (model M1) and all subsets except of the splice sites and young variants. In these subsets DD showed a higher *trait-specific per variant* ${h}^{2}$ (Fig. 1, Table S2).

**Fig. 2:**
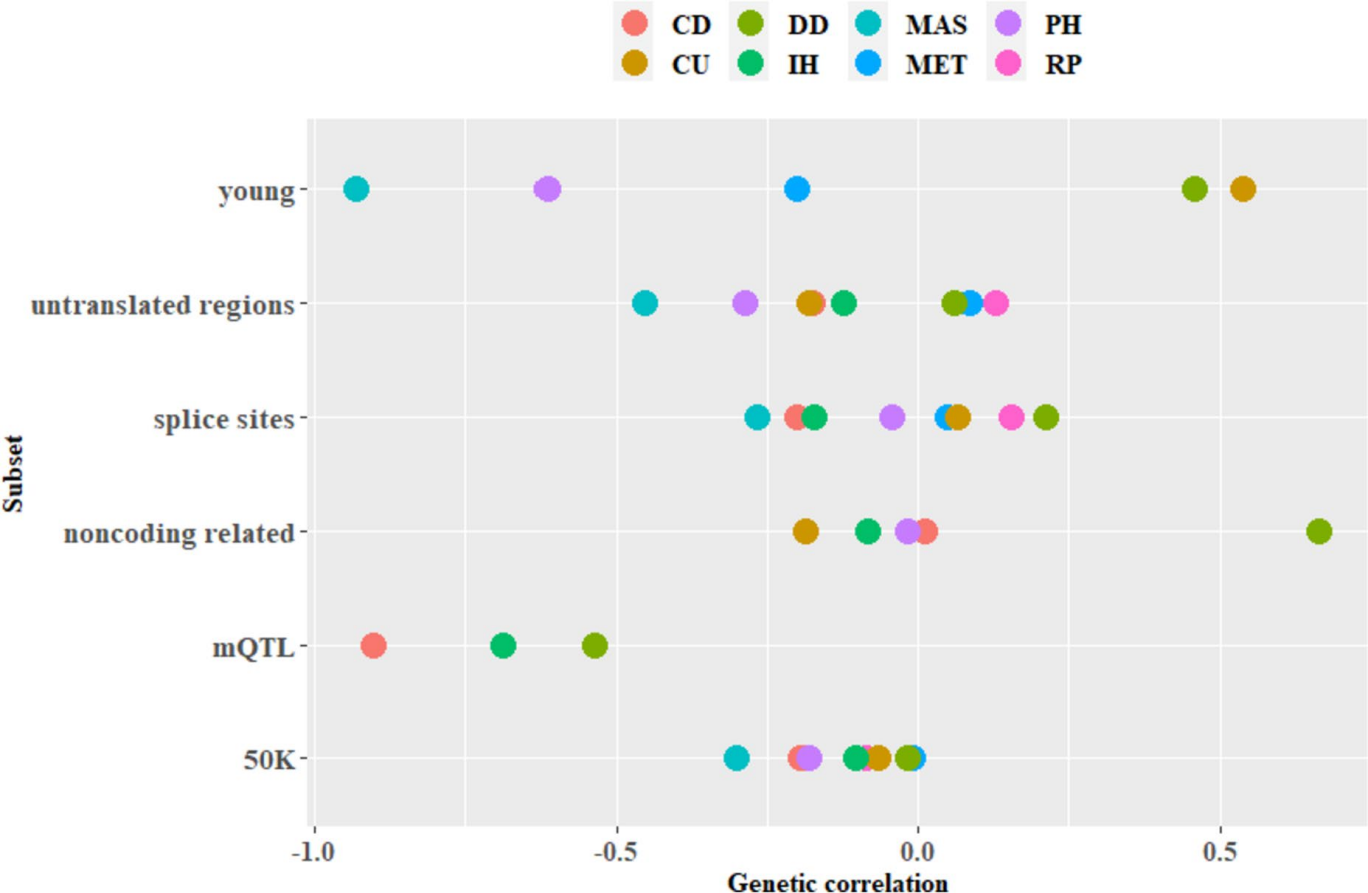
Genetic correlations between milk yield and the respective health traits. The health traits are cyclus disturbances (CD), retained placenta (RP), metritis (MET), mastitis (MAS), digital dermatitis (DD), interdigital hyperplasia (IH), digital phlegmon (PH), and claw ulcers (CU). Subset genetic correlations from model M4 were shown for the subsets (mQTL noncoding related, splice sites, untranslated regions, young) and genetic correlations from model M2 for the 50K chip

All genetic correlations of the 50K chip from model M2 were negative, ranging from -0.0111 between MY and MET to -0.3030 between MY and MAS (Table 1). For the subsets, we found that most ${r}_{g, set}$ estimates were negative. For DD, all subset genetic correlations except of the one for the mQTL were positive. For the mQTL subset, all estimates were negative. The strongest ${r}_{g, set}$ was found in the young subset (-0.9324, MY-MAS) and the weakest one between MY and CD in the noncoding related subset (0.0101). In general, the standard errors of the genetic correlations were considerable for the subsets, i.e., between 0.0841 (MY-DD) in the UTR subset and 0.8495 (MY-DD) in the noncoding related subset (Table S3). Conversely, they ranged from 0.0261 (MY-MET) to 0.0387 (MY-MET) for the 50K chip (Table 1). In this study, we defined ${r}_{g, set}$ estimates to be significant if they were at least two times higher than the corresponding standard error. Following this definition, two estimates in the subsets and all estimates for the 50K chip were significant. Both significant correlations were found in the UTR subset. They were between MY and PH (-0.2885, se = 0.1186) as well as MAS (-0.4558, se = 0.1070) (Fig. 2, Table S3).

The highest *trait-specific per variant* ${r}_{g}$ was observed between MY and DD ($2.089*{10}^{-4}$) in the noncoding related subset. In contrast to the univariate analysis, where the 50K chip ranked highest in terms of the *across trait* and *trait-specific per variant* ${h}^{2}$, we found that it had the lowest *trait-specific per variant* ${r}_{g}$ ($2.516*{10}^{-7}$ between MY and MET) (Table S4). While estimating the genetic correlations, not all trait combinations in all subsets converged. For the 50K chip, the UTR, and splice sites subset, all models did. However, for the noncoding related and young variants only five and for the mQTL subset only three models did converge (Tables S3 and S4).

**Fig. 3:**
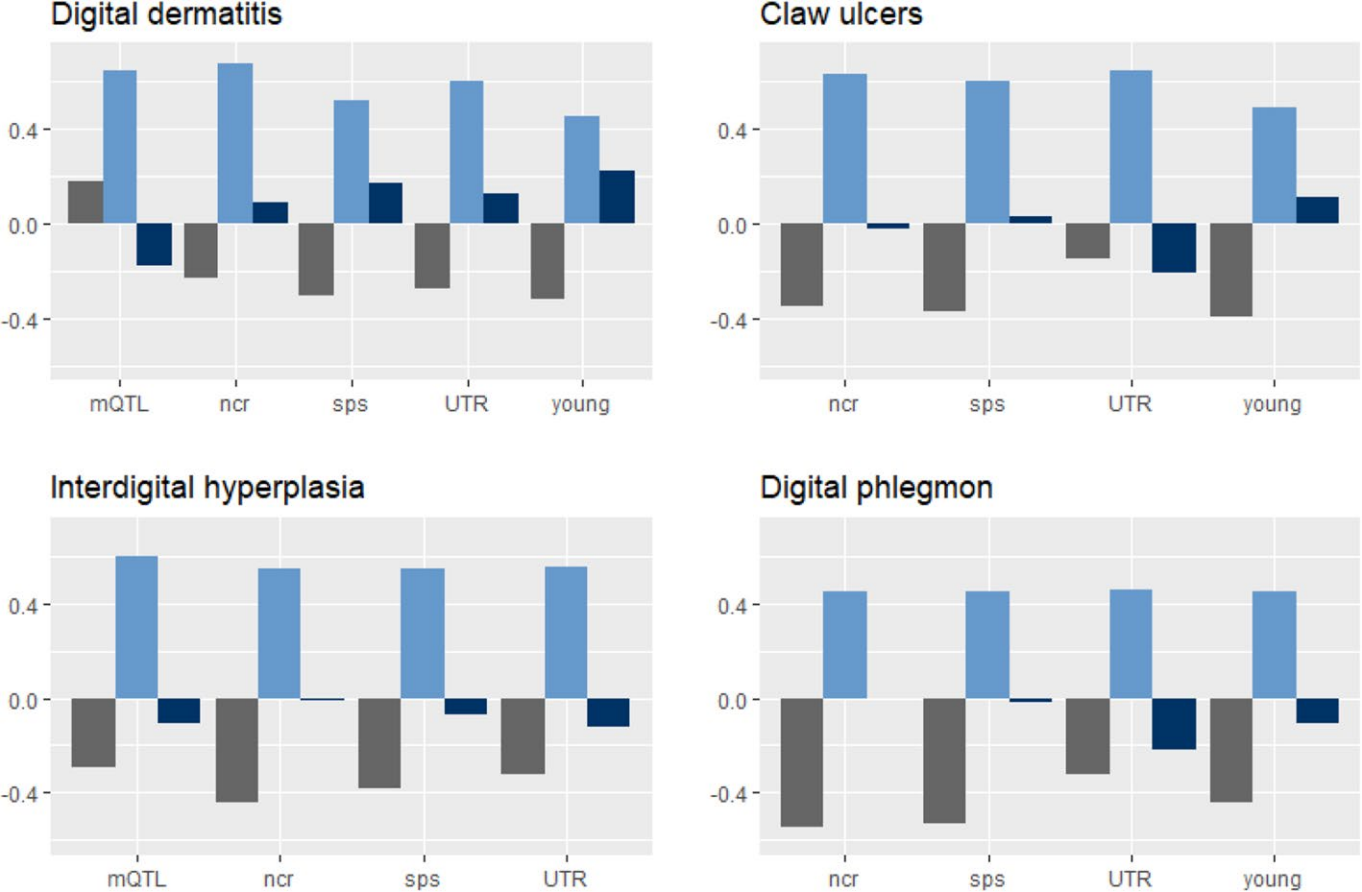
Relative covariance between milk yield and four claw health traits. Shown are the covariance terms of the 50K chip (grey bar), the residuum (lightblue bar), and the respective subset (darkblue bar) from model M4 for the subsets containing mQTL, noncoding related (ncr), splice sites (sps), untranslated regions (UTR), and young variants. Subsets for which the model did not converged are not shown. The relative covariance of each term was calculated as the respective covariance divided by the phenotypic covariance ($\sum(\left|{cov}_{50K}\right|+\left|{cov}_{set}\right|+\left|{cov}_e\right|)$)

**Fig. 4:**
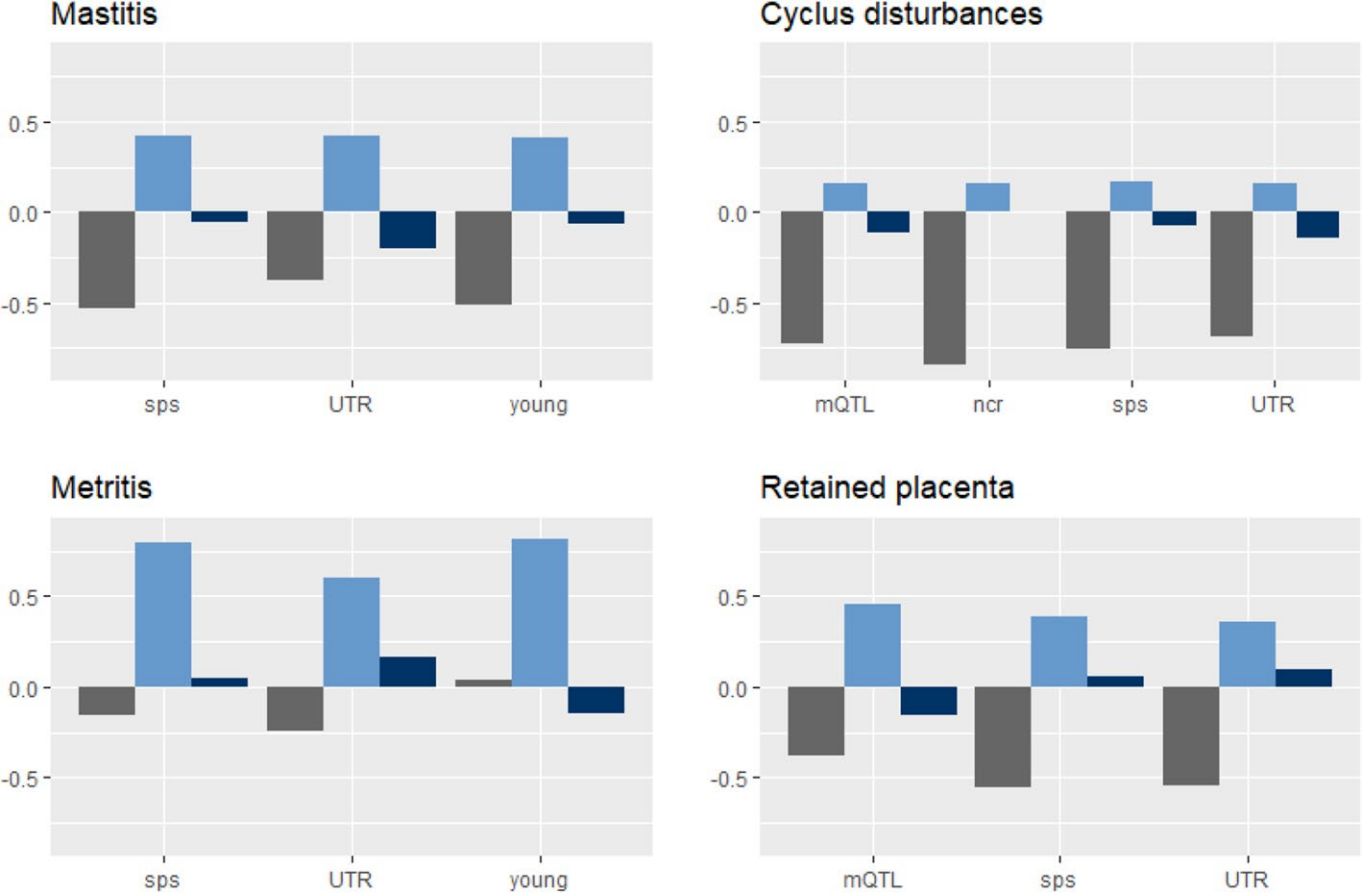
Relative covariance between milk yield and mastitis as well as three reproduction health traits. Shown are the covariance terms of the 50K chip (grey bar), the residuum (lightblue bar), and the respective subset (darkblue bar) from model M4 for the subsets containing mQTL, noncoding related (ncr), splice sites (sps), untranslated regions (UTR), and young variants. Subsets for which the model did not converged are not shown. The relative covariance of each term was calculated as the respective covariance divided by the phenotypic covariance ($\sum(\left|{cov}_{50K}\right|+\left|{cov}_{set}\right|+\left|{cov}_e\right|)$)

Moreover, we found the lowest ${relcov}_{50K}$ (0.0405) between MY and MET in the subset containing young variants, which was strongest between MY and CD in the noncoding related subset (-0.8406). Here, also ${relcov}_{set}$ was lowest with -0.0014. The young variants had the highest value of ${relcov}_{set}$ (0.2215) between MY and DD. The values of both parameters were positive as well as negative. Concerning the residual term, all values were positive and between 0.8127 for the young subset (between MY and MET) and 0.1580 for the noncoding related variants (between MY and CD). In general, ${relcov}_{50K}$ was stronger than ${relcov}_{set}$. However, ${relcov}_{set}$ was even larger or almost as large as ${relcov}_{50K}$ between MY and CU in the UTR subset and between MY and MET in the subset containing young variants. In most cases, the residual term explained most covariance (Figs. 3 and 4, Tables S5 to S12).

The overall genetic covariance explained by both polygenic terms, calculated as the sum of ${relcov}_{50K}$ and ${relcov}_{set}$, differed from the genetic covariance that was estimated using model M2. In contrast, for each trait the overall genetic variance (${h}_{set}^{2}+{h}_{50K}^{2}$) was equivalent to the heritability estimate from model M1 (results not shown). An overview over the absolute and relative values for the covariances and covariance parameters can be taken from the supplementary data (Tables S5 to S12).

### LD analysis

The mean *mean ld extern* was 0.2257, ranging from 0.1103 (LD1) to 0.3827 (LD3). Apart from the LD quartiles, the lowest *mean ld extern* was 0.1700 in the mQTL and 0.2738 in the VD4 subset. The mean *decay extern* was 0.4388, indicating that on average 43.88% of the LD between 0 and 25 kbp distance from a variant is still present between 120 and 500 kbp. Here the minimum was at 0.3066 (LD4) or 0.3659 (VD2) and the maximum 0.7651 (LD1) or 0.6486 (mQTL).

The mean *mean ld intern* was 0.2450, ranging from 0.0867 (conserved sites) to 0.8458 (selection signatures). Concerning the *decay intern*, the mean was 0.3791. Here, the minimum was 0.0741 in the LD4 subset, followed by 0.1253 in the noncoding related variants. The maximum *decay intern* was at 2.0580 in the LD1 subset, which indicates that an increasing physical distance between two variants in this very small window evokes an increased LD. However, the *mean LD intern* for the LD1 subset was low with 0.0958. The next highest subset was the LD2 subset (0.9651) followed by the MAF4 subset (0.5273). A detailed overview over all LD parameters can be taken from the supplementary data (Table S13).

Both, the *mean ld extern* (-0.1670) and the *mean ld intern* (-0.0484) were negatively correlated with the *across trait per variant* ${h}^{2}$ in a low to moderate range. This means that a lower LD inside and outside the subset variants induces that a subset explains more variance of the observed traits. However, the correlation between the *across trait per variant* ${h}^{2}$ and the *decay extern* was positive with 0.1143, which leads to the assumption that a less sharp decay outside the subset results in a higher *across trait per variant* ${h}^{2}$. The correlation to the *decay intern* is negative and low with -0.0231.

On average, the *mean MAF* was 0.2076, ranging from 0.1185 in the selection signatures subset to 0.3927 in the MAF4 subset. Here, the correlation to the *across trait per variant* ${h}^{2}$ was low and positive (0.1163), which means that a higher MAF results in a higher *across trait per variant* ${h}^{2}$.

The last parameter is the *distribution* with a mean of 0.9917, ranging from 0.9651 (mQTL) to 0.9980 (splice sites). A higher value indicates a rather equal distribution of the subset variants across the genome. This parameter’s correlation to the *across trait per variant* ${h}^{2}$ was moderate and negative with -0.1181, indicating that variants in rather accumulated regions explain more variance than variants in unique spots on the genome.

## Discussion

Current estimates of heritabilities and genetic correlation of milk production and disease traits in cattle are mostly derived from variance component estimations using either pedigrees or 50K chip genotypes. However, recent studies incorporating functional information about sequence variants in genomic analyses enhanced the understanding of molecular and biological mechanisms underlying complex traits and their genetic connection in cattle [18, 20, 25]. Further, they demonstrated benefits for the power to detect causal mutations and the accuracy of GP [14, 20, 28, 45]. At this, accuracies are enhanced especially for small populations and across-breed predictions, generally suffering from low accuracies [14, 26]. Conversely, populations that are already having high prediction accuracies using the common 50K chip show only little or no advantage when functional information is included in GP [29]. Up to date, a bivariate analysis of economically important cattle traits incorporating functional information has not been performed, to our best knowledge. Thus, we aimed at filling this gap with this study. Our results identify subsets of variants with a noticeable contribution to the genetic connection between milk yield and health traits in German Holstein cattle. In addition, they revealed that the subset genetic correlations were not only negative but also positive and that the high-ranking subsets’ effect does not seem to be induced by the LD structure between the subset variants or their LD with adjacent sequence variants. Nevertheless, the results of this study should be considered with caution since most of the subsets’ estimates standard errors are remarkable.

### Variance component estimation

The subsets’ *across trait per variant* ${h}^{2}$ in our study (Table 2) are similar to the results of Xiang et al. [20] and the heritabilities of the 50K chip from model M1 (Table 1) are in agreement with other studies based on pedigree data [30, 46]. Also, the negative genetic correlations from model M2 (Fig. 2, Table 1) in this study are in agreement with previous studies [30, 47, 48]. Whereas we applied the 50 K chip in this study, Xiang et al. [20] applied the denser HD chip. We justified our choice with the fact that the study of Xiang et al. [20] analyzed a multibreed dataset, which evokes a reduced LD and requires a denser marker panel to account for the reduced LD. Since we examine only one breed in this study, German Holstein, we assume that the 50K chip was sufficient to account for LD among the common and not causal variants.

Concerning the results from models M3 and M4, it is important to keep in mind that we applied two GRMs. This means that the subset heritabilities were meant to indicate which subsets explain additional variance to the one that is already explained by the 50K GRM. In fact, we found that the overall genetic variance (${h}_{set}^{2}+$ ${h}_{50K}^{2}$) (Table S1) did not differ from the heritabilities that were estimated using only the 50K chip (Table 1). This was somewhat surprising since Haile-Mariam et al. [49] supposed that the 50K chip underestimates the genetic variance of complex traits. When switching to the genetic covariance between milk yield and health traits, we found that the genetic covariance from model M2 solely based on chip data did not match the overall genetic covariance explained by both polygenic terms from model M4. Even though the genetic covariance explained by the 50K chip exceeds the one explained by the subsets in almost each case (Figs. 3 and 4, Tables S5 to S12), the subsets seem to provide additional information about the direction of the shared effect between traits (Fig. 2). However, it has to be kept in mind that most standard errors of the subsets’ estimates were considerable (Tables S3, S5 to S12).

The higher *trait-specific per variant* ${r}_{g}$ of the subsets in relation to the one from the 50K chip (Table S4) and the novel information they revealed in terms of the genetic covariance are probably related to the lack of causal variants on the 50K chip. Causal variants are most likely pleiotropic [14], and pleiotropy is one of the mechanisms underlying genetic correlations. Thus, applying the sequence variants that are very likely to be either causal themselves or in higher LD with a causal variant reveals more information, e.g., about the extent of the shared effect. This is because they capture the causal effect more precisely than via LD as the 50K chip does.

The findings of this study indicate a noteworthy amount of genetic covariance between milk yield and health traits in cattle that can be assigned to various subsets of functionally and evolutionary relevant genome partitions. Previous studies [14, 25–27] reported the advantages of models including functional information in GP to improve prediction accuracy by outperforming LD between causal and genotyped variants. Thus, our results address the potential to include causal variants in genomic prediction with the aim to capture the genetic correlation between milk production and disease traits more precisely. What makes it particularly attractive, is the fact that Cai et al. [50] introduced a different weighting of variants based on their pleiotropic effect which for example increases milk yield but decreases mastitis resistance. By weighting these variants differently in GP, it might be possible to minimize the unfavorable effect of high production on animal health and welfare. Since our results reveal subsets, whose covariance between milk yield and dairy health is positive, we assume that incorporating these variants with a different weight would enhance current cattle breeding.

Xiang et al. [14] increased the number of causal variants for GP by incorporating variants with a pleiotropic effect and functional significance, which lead to an increased prediction accuracy. They developed a new 65K genotyping array consisting of around 40% non-intergenic variants such as UTR, splice sites, and noncoding related variants, around 30% regulatory variants such as mQTL and sQTL and 5% variants that are involved in evolutionary processes, within or across species, including selection signatures and young variants. Thereby, all high-ranking subsets in our study are represented on the new 65K chip, which performed as good in prediction accuracy as much denser genotyping chips [14].

However, some standard errors were noteworthy, especially those of the subset genetic correlations. Here, also not all models did converge (Table S3). We attribute this to the small number of variants in some subsets. It would be interesting to perform follow-up analyses mapping the signals of genomic connection between milk production and health traits in more detail, for instance by applying tools to detect shared genomic regions as done in Schneider et al. [30]. This can be combined with functional and evolutionary information to scrutinize the role of these regions in transcriptional and translational processes and their evolutionary background.

### Biological and molecular mechanisms

The importance of UTR variants in our study can be supported by findings in human studies that attribute a strong association with various and especially disease traits to the 3´ UTR [51]. UTR, as well as noncoding related and gene end variants, are part of the cis-regulatory variants [52] altering translation efficiency, which leads to a differential gene expression.

Other regulatory elements are intermediate QTL, namely geQTL, eeQTL, aseQTL, and sQTL. Their importance for complex trait variation has repeatedly been shown [18, 24, 36, 53]. Moreover, it is generally assumed that differential gene expression is one of the main drivers of variation in quantitative traits [53–55]. However, whereas the rank of intermediate QTL was high in the study of Xiang et al. [20], their contribution to the trait variation in our study was negligible. On one side, we attribute this discrepancy to differences in the population structures between the populations used for the discovery analysis of intermediate QTL and the population used for the variance component estimation in our study. While the discovery analysis was carried out on Australian Holstein, Jersey, and Angus, our study is based on German Holstein. Several authors have reported different LD structures of Holstein Friesian populations around the world [56–58]. Moreover, LD varies between breeds as found by Gibbs et al. [59]. Thus, some QTL chosen in the discovery analyses [20, 24] might not be the causal variants but capture their effect via LD. In this case, QTL are not informative anymore in our study because of the different LD structure. Further, there are several factors that induce a lack of power in the detection of intermediate QTL, which might be the reason for the negligible effect of these QTL in this study. One factor is that, even though their effect is very consistent across tissues [18, 24], their activity might follow physiological and developmental changes of the animal. Hence, it is inevitable to sample the right tissue at the right time for a precise inference [36]. In this study, we applied intermediate QTL taken from discovery analyses based on liver and muscle tissue from Angus steers or white blood and milk cells from lactating cows [23, 24, 37]. Thus, it is possible that these intermediate QTL are different from those affecting the health traits in this study, which would explain the low effect that we observed. It is also important to mention that the overlap between variants in the dataset of Xiang et al. [20] and our analysis is only about 13 million variants. Therefore, highly important QTL without an overlap with our dataset might have been lost.

As mentioned above, intermediate QTL play an important role in the genetic variation of complex traits. They are said to be enriched in UTR [24]. As well, sQTL, belonging to the group of intermediate QTL, have a high overlap with splice variants [18]. Hence, we believe that these findings support the high rank of UTR and splice site variants in our study. In a study on cattle data, Xiang et al. [53] found that sQTL alone explain as much variance as other regulatory QTL jointly, which highlights the importance of alternative splicing for phenotypic variation. This can be supported by the results from Wang et al. [60], who found that around 50% of differentially expressed genes for mastitis resistance showed alternative splicing. Interestingly, we found that the trait-specific *per variant* ${h}^{2}$ was highest for MY in all subsets but the splice sites, where DD ranked highest (Table S2). In general, the trait-specific *per variant* ${h}^{2}$ values were more alike in the splice sites subset than it was in others like the mQTL subset (Table S2). This supports the importance of alternative splicing for various complex traits.

The high rank of noncoding related variants in the univariate analysis is in agreement with Xiang et al. [20]. They can be split into two different categories, the small noncoding RNAs (sncRNA) and long noncoding RNAs (lncRNA). sncRNAs play an important role in the regulation of gene expression via post-transcriptional modification and splicing [61]. A subgroup of the sncRNAs are micro RNAs (miRNA), which have been found to be central for oncogenesis in humans [61] and to affect the bovine physiology and development [62, 63]. Just like the sncRNAs, lncRNAs affect RNA splicing as well [64]. In addition, they were identified as key regulators of the energy metabolism and lipogenesis in mammals [65–67]. Also in humans, they have been shown to be related to metabolic disorders like obesity [68–70]. This confirms the connection of milk production and health in dairy cattle via the animals’ metabolic burden.

mQTL are defined as QTL altering the concentration of 19 bovine milk fat polar lipids [20], which strongly depends on the total amount of milk fat [71]. The latter increases during times where the animal experiences a negative energy balance (NEB) [72]. Thus, mQTLs might reflect, to some extent, the animals’ body fat mobilization, which is highest in the early lactational NEB when the cow is most susceptible to diseases [73]. Xiang et al. [20] reported a high impact of mQTL as well. Many of the traits they analysed in their study are milk production traits, which are very likely to be affected by the mQTL. By calculating the trait-specific *per variant* ${h}^{2}$ we were able to investigate the effect of this subset in more detail and found that it was in fact highest for MY ($1.871*{10}^{-5}$). Nevertheless, the *trait-specific per variant* ${h}^{2}$ for MAS was with $1.178*{10}^{-6}$ almost as high as the one for MY (Table S2)*.* Variants of the mQTL subset are enriched in and around *DGAT1* [20], a major QTL for milk production [74, 75]. Another highly important QTL for milk production is the gene *MGST1*, which is located on chromosome 5. However, no variant in the mQTL subset is located on chromosome 5. Thus, it seems like mQTL do not only have an effect on milk fat synthesis. Moreover, they might affect body functions in tissues other than the mammary gland as well, putatively related to general processes of the lipid mobilization and synthesis [20]. It has to be noted, that previous studies already mentioned the effect of *DGAT1* on milk yield, udder health, and fertility in inverse directions [30, 50, 76].

### LD analysis

While analyzing the subsets’ *across trait per variant* ${h}^{2}$ and the correlation with their internal and external LD structure, their distribution over the genome and their MAF, we aimed to dissect whether high ranking subsets might indeed harbor potential causal mutations or if their effect is merely based on linkage between variants, their MAF or their accumulated effect.

The LD decay in our study is in line with other studies, observing decreasing LD with increasing physical distance using both, medium density and sequence data [57, 77, 78]. We did not aim to deeply scrutinize the LD structure, phase consistency and other LD properties of the observed population. Thus, we will not go into more detail about those population specific parameters.

Our first hypothesis was that a positive correlation between the *mean ld extern* and the *across trait per variant* ${h}^{2}$ gives a hint that the 50K chip did not account properly for extensive LD in the surrounding variants. However, the actual correlation was moderate and negative with -0.1670, which indicates that by incorporating the 50K chip, extensive LD upward biasing the variance explained by the subset variants was diminished. This is supported by the results of Xiang et al. [20], where the higher LD variants did not have a higher *across trait per variant* ${h}^{2}$.

Next, we investigated the LD structure within the subset variants. This was done to observe, whether a subset’s high effect is induced by the accumulated and LD based effect rather than by some causal variants having a strong effect. In fact, the correlation with the *mean ld intern* was low and negative (-0.0484) as well as the one to the *decay intern* (-0.0231). Hence, LD within the subsets does not induce an elevated *across trait per variant* ${h}^{2}$ of a subset.

However, the negative correlation between the *across trait per variant* ${h}^{2}$ and the *distribution* indicates that subset variants, which are more accumulated, explain more variance of the observed traits. Thus, we assume that variants in subsets explaining more variance are all having an impact, which does not arise because they are in high LD.

A possible explanation for this is the assumption that the marker effects follow a normal distribution in our model. This assumption, its limitation and solutions like the application of Bayesian models have been discussed in the literature (e.g., [28, 79, 80]). Our choice of normally distributed marker effects was nevertheless based on the increasing complexity coming along with Bayesian models. Even though our genotypic data contained ~17 million variants, some subsets consisted of only few thousands of variants (Table 2). This might hamper the accurate estimation of their effects while applying more complex models.

While observing the LD structure inside and outside the subset variants, we found some differences. Whereas the correlation between the *across trait per variant* ${h}^{2}$ and the *mean LD extern* is negative, the one to the *decay extern* is positive. This indicates that a high mean ${r}^{2}$ but also a rapid decay evokes a reduced *across trait per variant* ${h}^{2}$ of a subset. In contrast, there is no effect of *decay intern* on the *across trait per variant* ${h}^{2}$. Interestingly, others [81, 82] have previously reported differences between LD properties of intergenic and intragenic regions. The mean LD in intergenic regions is slightly higher and decays significantly more rapid than LD in intragenic regions [82].

Finally, there is a positive but low correlation between the *across trait per variant* ${h}^{2}$ and the subsets’ *mean MAF* (0.1163). This is in line with Xiang et al. [20] who did not find a strong influence of allele frequencies on the subsets’ *across trait per variant* ${h}^{2}$. Additionally, we found a high rank of the young variants in the uni- as well as the bivariate analysis, whose mean MAF is also high (0.2770). These variants are expected to be favorable in terms of recent selection, which is characterized by their low correlation with rare variants [20].

For some traits (CU and DD), the genetic correlations where positive, which is at least partly in agreement with the shift in breeding towards healthier cows during the last years. The latter findings are somewhat surprising since recent studies showed the importance of rare variants for health and fertility traits in cattle [83, 84]. However, it has to be noticed that rare variants have not been included in this study to prevent biased results due to inaccuracies in the imputation of rare variants [84]. Therefore, the importance of young variants and the correlation of the subsets’ *mean MAF* with the *across trait per variant* ${h}^{2}$ might change while including these variants in the analyses.

## Conclusion

In this study, the large sample size was utilized to elucidate the contribution of 27 genome partitioning subsets with functional and evolutionary information about ~17 million sequence variants to the genetic (co-)variance of milk yield and health traits in dairy cattle. Thereby, the aim was to identify subsets of sequence variants explaining additional genetic (co-)variance to the one explained by the 50K chip. In fact, the 50K chip was sufficient to explain the genetic variance and no subset provided new insights. However, the opposite was found in terms of the genetic covariance. Here, subsets were found that revealed new information about the extent and direction of the genetic connection between milk yield and the health traits. Their biological function and molecular mechanisms confirm the connection of the animal’s production and its health status via the negative energy balance and the importance of alternative splicing for complex trait variation. Both aspects have already been shown previously. Nevertheless, it has to be noted that most standard errors of the subsets estimates were remarkable. Further, our results show that these subsets’ high effect is very likely not erroneously upward biased by extensive LD in the cattle genome. This study indicates the potential of integrating functional information in GP to account for the covariance between economically important traits more precisely. Aiming at continuous improvements in cattle breeding, follow up studies are necessary that combine the detection of shared genomic regions with these regions’ functional annotation.

## Supplementary Information

**Supplementary Material 1.** 

## Data Availability

Restrictions apply to the availability of the genotype and phenotype data analyzed in this study since they are property of the German Holstein breeding organisations organized within the Bundesverband Rind und Schwein e.V.. We thank all participating organisations. Thus, the data have commercial value and are not publicly available. The corresponding author can be contacted for a reasonable request.
