# Supplementary material for: Genomic dissection of the correlation between milk yield and various health traits using functional and evolutionary information about imputed sequence variants of 34,497 German Holstein cows
Source: BMC Genomics. 2024 Mar 9;25:265. doi: 10.1186/s12864-024-10115-6 (PMC11385139; doi:10.1186/s12864-024-10115-6)
Supplement: Supplementary file 1 — Supplementary Material 1. [file 12864_2024_10115_MOESM1_ESM.docx]

**Table S1 Heritability estimates from model M3.**

| Trait | $h_{50K}^{2}$ | se$h_{50K}^{2}$ | $h_{set}^{2}$ | se $h_{set}^{2}$ | Subset |
| --- | --- | --- | --- | --- | --- |
| Cyclus disturbances | 0.0771 | 0.0055 | 0.0007 | 0.0018 | Conserved 100 |
| Metritis | 0.0558 | 0.0048 | 0.0019 | 0.0022 |  |
| Retained placenta | 0.0736 | 0.0053 | <0.0001 | 0.0019 |  |
| Digital dermatitis | 0.1735 | 0.0072 | <0.0001 | 0.0018 |  |
| Claw ulcers | 0.1525 | 0.0072 | <0.0001 | 0.0020 |  |
| Digital phlegmon | 0.0972 | 0.0062 | <0.0001 | 0.0019 |  |
| Interdigital hyperplasia | 0.1531 | 0.0069 | 0.0016 | 0.0019 |  |
| Mastitis | 0.1324 | 0.0063 | <0.0001 | 0.0014 |  |
| Milk yield | 0.4358 | 0.0078 | 0.0002 | 0.0010 |  |
| Cyclus disturbances | 0.0771 | 0.0055 | 0.0004 | 0.0018 | ChIPseq |
| Metritis | 0.0558 | 0.0048 | 0.0011 | 0.0020 |  |
| Retained placenta | 0.0737 | 0.0053 | <0.0001 | 0.0017 |  |
| Digital dermatitis | 0.1732 | 0.0072 | <0.0001 | 0.0017 |  |
| Claw ulcers | 0.1524 | 0.0072 | <0.0001 | 0.0019 |  |
| Digital phlegmon | 0.0970 | 0.0062 | <0.0001 | 0.0019 |  |
| Interdigital hyperplasia | 0.1531 | 0.0069 | 0.0014 | 0.0018 |  |
| Mastitis | 0.1324 | 0.0063 | <0.0001 | 0.0013 |  |
| Milk yield | 0.4357 | 0.0078 | <0.0001 | 0.0009 |  |
| Cyclus disturbances | 0.0329 | 0.0107 | 0.0513 | 0.0118 | LD1 |
| Metritis | 0.0195 | 0.0092 | 0.0425 | 0.0105 |  |
| Retained placenta | 0.0404 | 0.0104 | 0.0376 | 0.0113 |  |
| Digital dermatitis | 0.0975 | 0.0129 | 0.0859 | 0.0134 |  |
| Claw ulcers | 0.0590 | 0.0131 | 0.1044 | 0.0143 |  |
| Digital phlegmon | 0.0421 | 0.0109 | 0.0638 | 0.0119 |  |
| Interdigital hyperplasia | 0.0432 | 0.0116 | 0.1211 | 0.0129 |  |
| Mastitis | 0.0873 | 0.0112 | 0.0518 | 0.0115 |  |
| Milk yield | 0.3956 | 0.0140 | 0.0452 | 0.0128 |  |
| Cyclus disturbances | 0.0350 | 0.0136 | 0.0501 | 0.0142 | LD2 |
| Metritis | 0.0154 | 0.0125 | 0.0435 | 0.0132 |  |
| Retained placenta | 0.0366 | 0.0131 | 0.0391 | 0.0135 |  |
| Digital dermatitis | 0.0871 | 0.0159 | 0.0934 | 0.0163 |  |
| Claw ulcers | 0.0707 | 0.0169 | 0.0870 | 0.0173 |  |
| Digital phlegmon | 0.0404 | 0.0144 | 0.0615 | 0.0149 |  |
| Interdigital hyperplasia | 0.0676 | 0.0149 | 0.0916 | 0.0154 |  |
| Mastitis | 0.0671 | 0.0138 | 0.0702 | 0.0141 |  |
| Milk yield | 0.3236 | 0.0176 | 0.1216 | 0.0173 |  |
| Cyclus disturbances | 0.0537 | 0.0118 | 0.0245 | 0.0115 | LD3 |
| Metritis | 0.0414 | 0.0111 | 0.0151 | 0.0109 |  |
| Retained placenta | 0.0628 | 0.0114 | 0.0112 | 0.0108 |  |
| Digital dermatitis | 0.1237 | 0.0139 | 0.0542 | 0.0134 |  |
| Claw ulcers | 0.1117 | 0.0147 | 0.0430 | 0.0141 |  |
| Digital phlegmon | 0.0660 | 0.0129 | 0.0332 | 0.0126 |  |
| Interdigital hyperplasia | 0.1109 | 0.0132 | 0.0449 | 0.0127 |  |
| Mastitis | 0.1047 | 0.0122 | 0.0299 | 0.0116 |  |
| Milk yield | 0.3993 | 0.0150 | 0.0395 | 0.0138 |  |
| Cyclus disturbances | 0.0671 | 0.0080 | 0.0105 | 0.0064 | LD4 |
| Metritis | 0.0496 | 0.0075 | 0.0064 | 0.0062 |  |
| Retained placenta | 0.0774 | 0.0079 | <0.0001 | 0.0060 |  |
| Digital dermatitis | 0.1628 | 0.0096 | 0.0124 | 0.0070 |  |
| Claw ulcers | 0.1480 | 0.0099 | 0.0052 | 0.0073 |  |
| Digital phlegmon | 0.0837 | 0.0089 | 0.0147 | 0.0072 |  |
| Interdigital hyperplasia | 0.1514 | 0.0095 | 0.0016 | 0.0067 |  |
| Mastitis | 0.1252 | 0.0086 | 0.0081 | 0.0065 |  |
| Milk yield | 0.4456 | 0.0105 | <0.0001 | 0.0080 |  |
| Cyclus disturbances | 0.0504 | 0.0096 | 0.0276 | 0.0091 | MAF2 |
| Metritis | 0.0377 | 0.0087 | 0.0192 | 0.0084 |  |
| Retained placenta | 0.0632 | 0.0095 | 0.0108 | 0.0085 |  |
| Digital dermatitis | 0.1440 | 0.0118 | 0.0328 | 0.0107 |  |
| Claw ulcers | 0.1192 | 0.0125 | 0.0356 | 0.0116 |  |
| Digital phlegmon | 0.0838 | 0.0106 | 0.0149 | 0.0095 |  |
| Interdigital hyperplasia | 0.1107 | 0.0116 | 0.0454 | 0.0110 |  |
| Mastitis | 0.1071 | 0.0102 | 0.0282 | 0.0093 |  |
| Milk yield | 0.4157 | 0.0129 | 0.0233 | 0.0115 |  |
| Cyclus disturbances | 0.0619 | 0.0111 | 0.0166 | 0.0102 | MAF3 |
| Metritis | 0.0452 | 0.0103 | 0.0111 | 0.0097 |  |
| Retained placenta | 0.0689 | 0.0106 | 0.0049 | 0.0094 |  |
| Digital dermatitis | 0.1194 | 0.0137 | 0.0593 | 0.0135 |  |
| Claw ulcers | 0.1199 | 0.0144 | 0.0354 | 0.0138 |  |
| Digital phlegmon | 0.0750 | 0.0123 | 0.0240 | 0.0226 |  |
| Interdigital hyperplasia | 0.1461 | 0.0129 | 0.0075 | 0.0117 |  |
| Mastitis | 0.1083 | 0.0119 | 0.0260 | 0.0112 |  |
| Milk yield | 0.3126 | 0.0175 | 0.1358 | 0.0180 |  |
| Cyclus disturbances | 0.0623 | 0.0106 | 0.0155 | 0.0097 | MAF4 |
| Metritis | 0.0487 | 0.0097 | 0.0071 | 0.0086 |  |
| Retained placenta | 0.0693 | 0.0101 | 0.0045 | 0.0089 |  |
| Digital dermatitis | 0.1320 | 0.0132 | 0.0463 | 0.0128 |  |
| Claw ulcers | 0.1316 | 0.0134 | 0.0229 | 0.0124 |  |
| Digital phlegmon | 0.0750 | 0.0115 | 0.0234 | 0.0106 |  |
| Interdigital hyperplasia | 0.1503 | 0.0123 | 0.0030 | 0.0109 |  |
| Mastitis | 0.0933 | 0.0114 | 0.0426 | 0.0111 |  |
| Milk yield | 0.3941 | 0.0151 | 0.0468 | 0.0147 |  |
| Cyclus disturbances | 0.0665 | 0.0071 | 0.0110 | 0.0053 | untranslated regions |
| Metritis | 0.0428 | 0.0064 | 0.0137 | 0.0052 |  |
| Retained placenta | 0.0672 | 0.0071 | 0.0062 | 0.0051 |  |
| Digital dermatitis | 0.1163 | 0.0092 | 0.0567 | 0.0078 |  |
| Claw ulcers | 0.1253 | 0.0096 | 0.0268 | 0.0073 |  |
| Digital phlegmon | 0.0751 | 0.0080 | 0.0229 | 0.0063 |  |
| Interdigital hyperplasia | 0.1231 | 0.0090 | 0.0312 | 0.0071 |  |
| Mastitis | 0.1063 | 0.0081 | 0.0264 | 0.0063 |  |
| Milk yield | 0.3211 | 0.0120 | 0.1094 | 0.0105 |  |
| Cyclus disturbances | 0.0416 | 0.0114 | 0.0386 | 0.0116 | VD1 |
| Metritis | 0.0281 | 0.0101 | 0.0306 | 0.0105 |  |
| Retained placenta | 0.0611 | 0.0111 | 0.0134 | 0.0107 |  |
| Digital dermatitis | 0.0834 | 0.0132 | 0.0974 | 0.0136 |  |
| Claw ulcers | 0.0889 | 0.0141 | 0.0690 | 0.0143 |  |
| Digital phlegmon | 0.0481 | 0.0119 | 0.0542 | 0.0122 |  |
| Interdigital hyperplasia | 0.0727 | 0.0127 | 0.0876 | 0.0131 |  |
| Mastitis | 0.0742 | 0.0118 | 0.0638 | 0.0120 |  |
| Milk yield | 0.1676 | 0.0163 | 0.2757 | 0.0169 |  |
| Cyclus disturbances | 0.0460 | 0.0122 | 0.0338 | 0.0125 | VD2 |
| Metritis | 0.0242 | 0.0112 | 0.0343 | 0.0117 |  |
| Retained placenta | 0.0500 | 0.0119 | 0.0251 | 0.0119 |  |
| Digital dermatitis | 0.1379 | 0.0143 | 0.0401 | 0.1391 |  |
| Claw ulcers | 0.0980 | 0.0149 | 0.0590 | 0.0149 |  |
| Digital phlegmon | 0.0611 | 0.0129 | 0.0399 | 0.0129 |  |
| Interdigital hyperplasia | 0.0915 | 0.0134 | 0.0668 | 0.0135 |  |
| Mastitis | 0.0979 | 0.0125 | 0.0377 | 0.0122 |  |
| Milk yield | 0.4112 | 0.0146 | 0.0275 | 0.0135 |  |
| Cyclus disturbances | 0.0674 | 0.0104 | 0.0106 | 0.0098 | VD3 |
| Metritis | 0.0325 | 0.0094 | 0.0245 | 0.0093 |  |
| Retained placenta | 0.0717 | 0.0103 | 0.0021 | 0.0096 |  |
| Digital dermatitis | 0.1526 | 0.0122 | 0.0245 | 0.0112 |  |
| Claw ulcers | 0.1226 | 0.0129 | 0.0322 | 0.012 |  |
| Digital phlegmon | 0.0775 | 0.0113 | 0.0222 | 0.0107 |  |
| Interdigital hyperplasia | 0.1183 | 0.0118 | 0.0370 | 0.0108 |  |
| Mastitis | 0.1130 | 0.0107 | 0.0215 | 0.0098 |  |
| Milk yield | 0.4480 | 0.0128 | <0.0001 | 0.0111 |  |
| Cyclus disturbances | 0.0654 | 0.0072 | 0.0125 | 0.0057 | VD4 |
| Metritis | 0.0530 | 0.0067 | 0.0031 | 0.0052 |  |
| Retained placenta | 0.0722 | 0.007 | 0.0016 | 0.0049 |  |
| Digital dermatitis | 0.1603 | 0.0089 | 0.0153 | 0.0062 |  |
| Claw ulcers | 0.1403 | 0.0094 | 0.0133 | 0.0068 |  |
| Digital phlegmon | 0.0826 | 0.0081 | 0.0160 | 0.0063 |  |
| Interdigital hyperplasia | 0.1427 | 0.0089 | 0.0108 | 0.0064 |  |
| Mastitis | 0.1246 | 0.008 | 0.0088 | 0.0056 |  |
| Milk yield | 0.4418 | 0.0097 | <0.0001 | 0.0065 |  |
| Cyclus disturbances | 0.0768 | 0.0055 | <0.0001 | 0.0016 | aseQTL |
| Metritis | 0.0550 | 0.0047 | <0.0001 | 0.0017 |  |
| Retained placenta | 0.0730 | 0.0053 | <0.0001 | 0.0015 |  |
| Digital dermatitis | 0.1739 | 0.0072 | <0.0001 | 0.0014 |  |
| Claw ulcers | 0.1527 | 0.0072 | <0.0001 | 0.0017 |  |
| Digital phlegmon | 0.0971 | 0.0062 | <0.0001 | 0.0017 |  |
| Interdigital hyperplasia | 0.1531 | 0.0069 | 0.0013 | 0.0015 |  |
| Mastitis | 0.1320 | 0.0063 | <0.0001 | 0.0012 |  |
| Milk yield | 0.4358 | 0.0078 | 0.0002 | 0.0008 |  |
| Cyclus disturbances | 0.0771 | 0.0055 | 0.0001 | 0.0016 | coding related |
| Metritis | 0.0558 | 0.0048 | 0.0008 | 0.0018 |  |
| Retained placenta | 0.0737 | 0.0053 | 0.0012 | 0.0018 |  |
| Digital dermatitis | 0.1742 | 0.0072 | <0.0001 | 0.0014 |  |
| Claw ulcers | 0.1526 | 0.0072 | <0.0001 | 0.0017 |  |
| Digital phlegmon | 0.0976 | 0.0062 | <0.0001 | 0.0017 |  |
| Interdigital hyperplasia | 0.1531 | 0.0069 | 0.0024 | 0.0018 |  |
| Mastitis | 0.1324 | 0.0063 | <0.0001 | 0.0012 |  |
| Milk yield | 0.4358 | 0.0078 | 0.0002 | 0.0009 |  |
| Cyclus disturbances | 0.0770 | 0.0055 | <0.0001 | 0.0016 | eeQTL |
| Metritis | 0.0556 | 0.0048 | <0.0001 | 0.0017 |  |
| Retained placenta | 0.0736 | 0.0053 | <0.0001 | 0.0015 |  |
| Digital dermatitis | 0.1732 | 0.0072 | <0.0001 | 0.0015 |  |
| Claw ulcers | 0.1529 | 0.0072 | <0.0001 | 0.0017 |  |
| Digital phlegmon | 0.0974 | 0.0062 | <0.0001 | 0.0017 |  |
| Interdigital hyperplasia | 0.1531 | 0.0069 | 0.0014 | 0.0016 |  |
| Mastitis | 0.1328 | 0.0063 | 0.0002 | 0.0012 |  |
| Milk yield | 0.4357 | 0.0078 | <0.0001 | 0.0008 |  |
| Cyclus disturbances | 0.0771 | 0.0055 | 0.0002 | 0.001 | geQTL |
| Metritis | 0.0558 | 0.0048 | 0.0010 | 0.0012 |  |
| Retained placenta | 0.0737 | 0.0053 | 0.0003 | 0.0011 |  |
| Digital dermatitis | 0.1736 | 0.0072 | <0.0001 | 0.001 |  |
| Claw ulcers | 0.1526 | 0.0072 | <0.0001 | 0.0011 |  |
| Digital phlegmon | 0.0971 | 0.0062 | <0.0001 | 0.0012 |  |
| Interdigital hyperplasia | 0.0153 | 0.0069 | 0.0014 | 0.0011 |  |
| Mastitis | 0.2326 | 0.0063 | 0.0003 | 0.0008 |  |
| Milk yield | 0.4357 | 0.0078 | 0.0004 | 0.0007 |  |
| Cyclus disturbances | 0.0771 | 0.0055 | 0.0007 | 0.0017 | gene end |
| Metritis | 0.0550 | 0.0047 | <0.0001 | 0.0017 |  |
| Retained placenta | 0.0738 | 0.0053 | 0.0004 | 0.0018 |  |
| Digital dermatitis | 0.1741 | 0.0072 | <0.0001 | 0.0015 |  |
| Claw ulcers | 0.1528 | 0.0072 | <0.0001 | 0.0017 |  |
| Digital phlegmon | 0.0977 | 0.0062 | <0.0001 | 0.0017 |  |
| Interdigital hyperplasia | 0.1531 | 0.0069 | 0.0020 | 0.0018 |  |
| Mastitis | 0.1324 | 0.0063 | <0.0001 | 0.0012 |  |
| Milk yield | 0.4357 | 0.0078 | <0.0001 | 0.0009 |  |
| Cyclus disturbances | 0.0613 | 0.0115 | 0.0172 | 0.0113 | intergenic |
| Metritis | 0.0359 | 0.0104 | 0.0213 | 0.0105 |  |
| Retained placenta | 0.0710 | 0.0112 | 0.0030 | 0.0107 |  |
| Digital dermatitis | 0.1640 | 0.0134 | 0.0118 | 0.0127 |  |
| Claw ulcers | 0.1326 | 0.0141 | 0.0222 | 0.0134 |  |
| Digital phlegmon | 0.0710 | 0.0121 | 0.0291 | 0.0119 |  |
| Interdigital hyperplasia | 0.1106 | 0.0130 | 0.0459 | 0.0126 |  |
| Mastitis | 0.1153 | 0.0116 | 0.0193 | 0.0109 |  |
| Milk yield | 0.4509 | 0.0139 | <0.0001 | 0.0127 |  |
| Cyclus disturbances | 0.0077 | 0.0055 | 0.0007 | 0.0018 | intron |
| Metritis | 0.0558 | 0.0048 | 0.0018 | 0.0020 |  |
| Retained placenta | 0.0734 | 0.0053 | <0.0001 | 0.0019 |  |
| Digital dermatitis | 0.1735 | 0.0072 | <0.0001 | 0.0018 |  |
| Claw ulcers | 0.1530 | 0.0072 | 0.0002 | 0.0019 |  |
| Digital phlegmon | 0.0976 | 0.0062 | <0.0001 | 0.0018 |  |
| Interdigital hyperplasia | 0.1531 | 0.0069 | 0.0019 | 0.0019 |  |
| Mastitis | 0.1323 | 0.0063 | <0.0001 | 0.0013 |  |
| Milk yield | 0.4357 | 0.0078 | <0.0001 | 0.0010 |  |
| Cyclus disturbances | 0.0767 | 0.0055 | 0.0004 | 0.0007 | mQTL |
| Metritis | 0.0558 | 0.0048 | <0.0001 | 0.0003 |  |
| Retained placenta | 0.0736 | 0.0053 | 0.0003 | 0.0006 |  |
| Digital dermatitis | 0.1734 | 0.0072 | 0.0009 | 0.0010 |  |
| Claw ulcers | 0.1534 | 0.0073 | <0.0001 | 0.0008 |  |
| Digital phlegmon | 0.0962 | 0.0062 | 0.0014 | 0.001 |  |
| Interdigital hyperplasia | 0.1507 | 0.0069 | 0.0014 | 0.001 |  |
| Mastitis | 0.1286 | 0.0063 | 0.0061 | 0.0026 |  |
| Milk yield | 0.3209 | 0.0093 | 0.0969 | 0.0145 |  |
| Cyclus disturbances | 0.0744 | 0.0059 | 0.0027 | 0.0025 | noncoding related |
| Metritis | 0.0556 | 0.0051 | 0.0003 | 0.002 |  |
| Retained placenta | 0.0748 | 0.0058 | <0.0001 | 0.0024 |  |
| Digital dermatitis | 0.1747 | 0.0075 | <0.0001 | 0.0024 |  |
| Claw ulcers | 0.1512 | 0.0072 | 0.0017 | 0.0027 |  |
| Digital phlegmon | 0.0944 | 0.0066 | 0.0038 | 0.0028 |  |
| Interdigital hyperplasia | 0.1532 | 0.0073 | <0.0001 | 0.0023 |  |
| Mastitis | 0.1318 | 0.0066 | 0.0008 | 0.0022 |  |
| Milk yield | 0.4209 | 0.0085 | 0.0152 | 0.0039 |  |
| Cyclus disturbances | 0.0768 | 0.0055 | <0.0001 | 0.0016 | sQTL |
| Metritis | 0.0553 | 0.0048 | <0.0001 | 0.0018 |  |
| Retained placenta | 0.0733 | 0.0053 | <0.0001 | 0.0016 |  |
| Digital dermatitis | 0.1741 | 0.0072 | <0.0001 | 0.0015 |  |
| Claw ulcers | 0.1530 | 0.0072 | 0.0001 | 0.0017 |  |
| Digital phlegmon | 0.0974 | 0.0062 | <0.0001 | 0.0018 |  |
| Interdigital hyperplasia | 0.1531 | 0.0069 | 0.0024 | 0.0017 |  |
| Mastitis | 0.1326 | 0.0063 | <0.0001 | 0.0012 |  |
| Milk yield | 0.4357 | 0.0078 | <0.0001 | 0.0009 |  |
| Cyclus disturbances | 0.0772 | 0.0055 | 0.0002 | 0.0005 | selection signatures |
| Metritis | 0.0559 | 0.0049 | 0.0003 | 0.0005 |  |
| Retained placenta | 0.0736 | 0.0053 | <0.0001 | 0.0004 |  |
| Digital dermatitis | 0.1747 | 0.0072 | 0.0001 | 0.0003 |  |
| Claw ulcers | 0.1527 | 0.0072 | <0.0001 | 0.0005 |  |
| Digital phlegmon | 0.0979 | 0.0062 | <0.0001 | 0.0004 |  |
| Interdigital hyperplasia | 0.1530 | 0.0069 | 0.0003 | 0.0003 |  |
| Mastitis | 0.1324 | 0.0063 | <0.0001 | 0.0003 |  |
| Milk yield | 0.4357 | 0.0078 | <0.0001 | 0.0002 |  |
| Cyclus disturbances | 0.0674 | 0.0071 | 0.0098 | 0.005 | splice sites |
| Metritis | 0.0489 | 0.0062 | 0.0074 | 0.0047 |  |
| Retained placenta | 0.0682 | 0.0068 | 0.0055 | 0.0047 |  |
| Digital dermatitis | 0.1288 | 0.0088 | 0.0440 | 0.0067 |  |
| Claw ulcers | 0.1324 | 0.0093 | 0.0192 | 0.0064 |  |
| Digital phlegmon | 0.0776 | 0.0078 | 0.0205 | 0.0058 |  |
| Interdigital hyperplasia | 0.1295 | 0.0085 | 0.0233 | 0.0059 |  |
| Mastitis | 0.1069 | 0.0077 | 0.0251 | 0.0055 |  |
| Milk yield | 0.4092 | 0.0099 | 0.0271 | 0.0066 |  |
| Cyclus disturbances | 0.0780 | 0.0076 | <0.0001 | 0.0054 | young |
| Metritis | 0.0488 | 0.0068 | 0.0071 | 0.0052 |  |
| Retained placenta | 0.0743 | 0.0072 | <0.0001 | 0.0049 |  |
| Digital dermatitis | 0.1457 | 0.0098 | 0.0301 | 0.0079 |  |
| Claw ulcers | 0.1447 | 0.0097 | 0.0089 | 0.0072 |  |
| Digital phlegmon | 0.0918 | 0.0085 | 0.0061 | 0.0062 |  |
| Interdigital hyperplasia | 0.1558 | 0.0090 | <0.0001 | 0.0060 |  |
| Mastitis | 0.1261 | 0.0080 | 0.0066 | 0.0054 |  |
| Milk yield | 0.4191 | 0.0104 | 0.0179 | 0.0076 |  |

Shown are the heritabilities of the respective subset ($h_{set}^{2})$ and the 50K chip in the analysis of this subset ($h_{50K}^{2})$ together with their standard errors (se)*.*

| Trait | *trait-specific per variant* $h^{2}$ | | | | | |
| --- | --- | --- | --- | --- | --- | --- |
|  | 50K | mQTL | noncoding related | splice sites | untranslated regions | young |
| Cyclus disturbances | $1.742*{10}^{-6}$ | $7.723*{10}^{-8}$ | $8.467*{10}^{-7}$ | $1.341*{10}^{-6}$ | $3.923*{10}^{-7}$ | $1.134*{10}^{-11}$ |
| Metritis | $1.265*{10}^{-6}$ | $1.931*{10}^{-10}$ | $9.407*{10}^{-8}$ | $1.013*{10}^{-6}$ | $4.866*{10}^{-7}$ | $8.050*{10}^{-8}$ |
| Retained placenta | $1.672*{10}^{-6}$ | $5.793*{10}^{-8}$ | $3.136*{10}^{-10}$ | $7.526*{10}^{-7}$ | $2.211*{10}^{-7}$ | $1.134*{10}^{-11}$ |
| Digital dermatitis | $3.595*{10}^{-6}$ | $1.738*{10}^{-7}$ | $1.931*{10}^{-10}$ | $6.021*{10}^{-6}$ | $2.022*{10}^{-6}$ | $3.413*{10}^{-7}$ |
| Claw ulcers | $3.467*{10}^{-6}$ | $1.931*{10}^{-10}$ | $5.331*{10}^{-7}$ | $2.627*{10}^{-6}$ | $9.558*{10}^{-7}$ | $1.009*{10}^{-7}$ |
| Digital phlegmon | $2.221*{10}^{-6}$ | $2.703*{10}^{-7}$ | $1.192*{10}^{-8}$ | $2.805*{10}^{-6}$ | $8.167*{10}^{-7}$ | $6.916*{10}^{-8}$ |
| Interdigital hyperplasia | $3.467*{10}^{-6}$ | $2.703*{10}^{-7}$ | $3.136*{10}^{-10}$ | $3.188*{10}^{-6}$ | $1.113*{10}^{-6}$ | $1.134*{10}^{-11}$ |
| Mastitis | $3.005*{10}^{-6}$ | $1.178*{10}^{-6}$ | $2.509*{10}^{-7}$ | $3.435*{10}^{-6}$ | $9.415*{10}^{-7}$ | $7.483*{10}^{-8}$ |
| Milk yield | $9.876*{10}^{-6}$ | $1.871*{10}^{-5}$ | $4.766*{10}^{-6}$ | $3.708*{10}^{-6}$ | $3.901*{10}^{-6}$ | $2.030*{10}^{-7}$ |

**Table S2 Trait-specific per variant** $\boldsymbol{h}^{\boldsymbol{2}}$ **of five subsets and the 50K chip.**

Shown are the five subsets that had the highest *across trait* *per variant* $h^{2}$.

**Table S3 Genetic correlation estimates from model M4.**

| Health trait | $r_{g,50K}$ | se $r_{g,50K}$ | $r_{g,set}$ | se $r_{g,set}$ | Subset |
| --- | --- | --- | --- | --- | --- |
| Cyclus disturbances | -0.1966 | 0.0502 | -0.1756 | 0.1607 | untranslated regions |
| Metritis | -0.0428 | 0.0606 | 0.0848 | 0.1437 |  |
| Retained placenta | -0.1327 | 0.0505 | 0.1283 | 0.2224 |  |
| Digital dermatitis | -0.0525 | 0.0428 | 0.0586 | 0.0841 |  |
| Claw ulcers | -0.0347 | 0.0421 | -0.1809 | 0.1178 |  |
| Digital phlegmon | -0.1429 | 0.0500 | -0.2885 | 0.1186 |  |
| Interdigital hyperplasia | -0.0971 | 0.0410 | -0.1250 | 0.1057 |  |
| Mastitis | -0.2513 | 0.0400 | -0.4558 | 0.1070 |  |
| Cyclus disturbances | -0.1252 | 0.0358 | -0.9039 | 0.6358 | mQTL |
| Metritis | * |  | * |  |  |
| Retained placenta | * |  | * |  |  |
| Digital dermatitis | 0.0244 | 0.0279 | -0.5378 | 0.3887 |  |
| Claw ulcers | * |  | * |  |  |
| Digital phlegmon | * |  | * |  |  |
| Interdigital hyperplasia | -0.0697 | 0.0288 | -0.6864 | 0.4512 |  |
| Mastitis | * |  | * |  |  |
| Cyclus disturbances | -0.2000 | 0.0362 | 0.0101 | 0.3745 | noncoding related |
| Metritis | * |  | * |  |  |
| Retained placenta | * |  | * |  |  |
| Digital dermatitis | -0.0275 | 0.0274 | 0.6662 | 0.8495 |  |
| Claw ulcers | -0.0651 | 0.0294 | -0.1875 | 0.4978 |  |
| Digital phlegmon | -0.1837 | 0.0339 | -0.0178 | 0.3100 |  |
| Interdigital hyperplasia | -0.1044 | 0.0284 | -0.0852 | 0.3532 |  |
| Mastitis | * |  | * |  |  |
| Cyclus disturbances | -0.1986 | 0.0435 | -0.2028 | 0.2514 | splice sites |
| Metritis | -0.0177 | 0.0488 | 0.0465 | 0.2822 |  |
| Retained placenta | -0.1122 | 0.0431 | 0.1550 | 0.3163 |  |
| Digital dermatitis | -0.0559 | 0.0348 | 0.2104 | 0.1386 |  |
| Claw ulcers | -0.0800 | 0.0350 | 0.0638 | 0.2047 |  |
| Digital phlegmon | -0.2073 | 0.0422 | -0.0434 | 0.1861 |  |
| Interdigital hyperplasia | -0.1018 | 0.0341 | -0.1724 | 0.1760 |  |
| Mastitis | -0.3141 | 0.0337 | -0.2672 | 0.1601 |  |
| Cyclus disturbances | * |  | * |  | young |
| Metritis | 0.0042 | 0.0500 | -0.2017 | 0.4184 |  |
| Retained placenta | * |  | * |  |  |
| Digital dermatitis | -0.0631 | 0.0343 | 0.4572 | 0.2475 |  |
| Claw ulcers | -0.0973 | 0.0344 | 0.5401 | 0.4568 |  |
| Digital phlegmon | -0.1595 | 0.0402 | -0.6170 | 0.4393 |  |
| Interdigital hyperplasia | * |  | * |  |  |
| Mastitis | -0.2792 | 0.0320 | -0.9324 | 0.6268 |  |

Shown are the genetic correlations of the respective subset ($r_{g,set})$ and the 50K chip in the analysis of this subset ($r_{g,50K})$ together with their standard errors (se)*.* A * denotes that the respective model did not converge.

| Health trait | *trait-specific per variant* $r_{g}$ | | | | | |
| --- | --- | --- | --- | --- | --- | --- |
|  | 50K | mQTL | noncoding related | splice sites | untranslated regions | young |
| Cyclus disturbances | -$4.464*{10}^{-6}$ | -$1.745*{10}^{-4}$ | $3.167*{10}^{-6}$ | -$2.775*{10}^{-5}$ | -$6.263*{10}^{-6}$ | * |
| Metritis | -$2.516*{10}^{-7}$ | * | * | $6.362*{10}^{-5}$ | $3.024*{10}^{-6}$ | -$2.287*{10}^{-6}$ |
| Retained placenta | -$1.990*{10}^{-6}$ | * | * | $2.120*{10}^{-5}$ | $4.576*{10}^{-6}$ | * |
| Digital dermatitis | -$4.238*{10}^{-7}$ | -$1.038*{10}^{-4}$ | $2.089*{10}^{-4}$ | $2.879*{10}^{-5}$ | $2.090*{10}^{-6}$ | $5.184*{10}^{-6}$ |
| Claw ulcers | -$1.552*{10}^{-6}$ | * | -$5.880*{10}^{-5}$ | $8.730*{10}^{-6}$ | -$6.452*{10}^{-6}$ | $6.124*{10}^{-6}$ |
| Digital phlegmon | -$4.115*{10}^{-6}$ | * | -$5.582*{10}^{-6}$ | -$5.939*{10}^{-6}$ | -$1.029*{10}^{-5}$ | -$6.996*{10}^{-6}$ |
| Interdigital hyperplasia | -$2.400*{10}^{-6}$ | -$1.325*{10}^{-4}$ | -$2.672*{10}^{-5}$ | -$2.359*{10}^{-5}$ | -$4.458*{10}^{-6}$ | * |
| Mastitis | -$6.867*{10}^{-6}$ | * | * | -$3.660*{10}^{-5}$ | -$1.626*{10}^{-6}$ | -$1.057*{10}^{-5}$ |

**Table S4 Trait-specific per variant** $\boldsymbol{r}_{\boldsymbol{g}}$ **of five subsets and the 50K chip.**

A * denotes that the respective model did not converge

**Table S5 Absolute and relative covariances between milk yield and digital dermatitis from model M4.**

| Subset | ${cov}_{set}$ | se ${cov}_{set}$ | ${relcov}_{set}$ | ${cov}_{50K}$ | se ${cov}_{50K}$ | ${relcov}_{50K}$ | ${cov}_{e}$ | se ${cov}_{e}$ | ${relcov}_{e}$ |
| --- | --- | --- | --- | --- | --- | --- | --- | --- | --- |
| young | 209.8721 | 107.9772 | 0.2215 | -305.4540 | 165.4515 | -0.3223 | 432.2604 | 88.8814 | 0.4562 |
| untranslated regions | 89.6264 | 128.4470 | 0.1241 | -197.6069 | 161.0761 | -0.2736 | 430.8925 | 88.7228 | 0.6024 |
| splice sites | 142.8437 | 93.6877 | 0.1732 | -250.8633 | 155.9298 | -0.3042 | 430.8925 | 88.7958 | 0.5225 |
| noncoding related | 56.3274 | 45.6440 | 0.0892 | -145.6713 | 145.3083 | -0.2307 | 429.4480 | 88.6981 | 0.6801 |
| mQTL | -114.4619 | 72.4622 | -0.1808 | 111.2313 | 127.1435 | 0.1757 | 407.4910 | 88.3672 | 0.6436 |

Shown is the residual covariance (${cov}_{e}$), the genetic covariance of the respective subset (${cov}_{set})$ and the corresponding 50K chip genetic covariance (${cov}_{50K}$) together with the standard errors (se) of these estimates. Also shown is the relative covariance explained by these three terms (${relcov}_{set}$*,* ${relcov}_{50K}$*,* ${relcov}_{e}$).

**Table S6 Absolute and relative covariances between milk yield and interdigital hyperplasia from model M4.**

| Subset | ${cov}_{set}$ | se ${cov}_{set}$ | ${relcov}_{set}$ | ${cov}_{50K}$ | se ${cov}_{50K}$ | ${relcov}_{50K}$ | ${cov}_{e}$ | se ${cov}_{e}$ | ${relcov}_{e}$ |
| --- | --- | --- | --- | --- | --- | --- | --- | --- | --- |
| untranslated regions | -91.4199 | 77.5900 | -0.1237 | -239.6552 | 101.3440 | -0.3243 | 407.8078 | 56.5219 | 0.5519 |
| splice sites | -54.8305 | 55.8494 | -0.0721 | -292.3583 | 97.9085 | -0.3847 | 412.8445 | 56.5556 | 0.5432 |
| noncoding related | -7.0353 | 29.1694 | -0.0095 | -328.0333 | 89.6293 | -0.4425 | 406.2000 | 56.3658 | 0.5480 |
| mQTL | -70.2288 | 39.4833 | -0.1091 | -190.3926 | 78.9923 | -0.2957 | 383.3065 | 56.3261 | 0.5953 |

Shown is the residual covariance (${cov}_{e}$), the genetic covariance of the respective subset (${cov}_{set})$ and the corresponding 50K chip genetic covariance (${cov}_{50K}$) together with the standard errors (se) of these estimates. Also shown is the relative covariance explained by these three terms (${relcov}_{set}$*,* ${relcov}_{50K}$*,* ${relcov}_{e}$).

**Table S7 Absolute and relative covariances between milk yield and retained placenta from model M4.**

| Subset | ${cov}_{set}$ | se ${cov}_{set}$ | ${relcov}_{set}$ | ${cov}_{50K}$ | se ${cov}_{50K}$ | ${relcov}_{50K}$ | ${cov}_{e}$ | se ${cov}_{e}$ | ${relcov}_{e}$ |
| --- | --- | --- | --- | --- | --- | --- | --- | --- | --- |
| untranslated regions | 63.1487 | 105.1448 | 0.0928 | -370.6178 | 141.1672 | -0.5447 | 246.6802 | 93.6082 | 0.3625 |
| splice sites | 38.7892 | 77.4199 | 0.0603 | -355.5203 | 136.0232 | -0.5529 | 248.6449 | 93.6841 | 0.3867 |
| mQTL | -85.4975 | 49.5863 | -0.1585 | -206.9850 | 106.7721 | -0.3838 | 246.8854 | 93.2388 | 0.4577 |

Shown is the residual covariance (${cov}_{e}$), the genetic covariance of the respective subset (${cov}_{set})$ and the corresponding 50K chip genetic covariance (${cov}_{50K}$) together with the standard errors (se) of these estimates. Also shown is the relative covariance explained by these three terms (${relcov}_{set}$*,* ${relcov}_{50K}$*,* ${relcov}_{e}$).

**Table S8 Absolute and relative covariances between milk yield and cyclus disturbances from model M4.**

| Subset | ${cov}_{set}$ | se ${cov}_{set}$ | ${relcov}_{set}$ | ${cov}_{50K}$ | se ${cov}_{50K}$ | ${relcov}_{50K}$ | ${cov}_{e}$ | se ${cov}_{e}$ | ${relcov}_{e}$ |
| --- | --- | --- | --- | --- | --- | --- | --- | --- | --- |
| untranslated regions | -124.5827 | 114.2678 | -0.1470 | -585.7482 | 151.8649 | -0.6910 | 137.3801 | 101.6341 | 0.1621 |
| splice sites | -69.0601 | 85.6860 | -0.0777 | -673.0017 | 148.3937 | -0.7571 | 146.908 | 101.6952 | 0.1653 |
| noncoding related | 1.2249 | 45.5724 | 0.0014 | -727.6464 | 134.4906 | -0.8406 | 136.7933 | 101.6504 | 0.1580 |
| mQTL | -96.2237 | 54.1445 | -0.1121 | -623.1029 | 116.8925 | -0.7261 | 138.7932 | 101.1788 | 0.1617 |

Shown is the residual covariance (${cov}_{e}$), the genetic covariance of the respective subset (${cov}_{set})$ and the corresponding 50K chip genetic covariance (${cov}_{50K}$) together with the standard errors (se) of these estimates. Also shown is the relative covariance explained by these three terms (${relcov}_{set}$*,* ${relcov}_{50K}$*,* ${relcov}_{e}$).

**Table S9 Absolute and relative covariances between milk yield and metritis from model M4.**

| Subset | ${cov}_{set}$ | se ${cov}_{set}$ | ${relcov}_{set}$ | ${cov}_{50K}$ | se ${cov}_{50K}$ | ${relcov}_{50K}$ | ${cov}_{e}$ | se ${cov}_{e}$ | ${relcov}_{e}$ |
| --- | --- | --- | --- | --- | --- | --- | --- | --- | --- |
| young | -41.8675 | 84.3867 | -0.1468 | 11.5528 | 135.9644 | 0.0405 | 231.7225 | 94.3881 | 0.8127 |
| untranslated regions | 61.6774 | 103.9526 | 0.1591 | -94.6507 | 133.7698 | -0.2442 | 231.2565 | 94.2778 | 0.5967 |
| splice sites | 12.5618 | 76.3909 | 0.0430 | -47.2942 | 130.2330 | -0.1618 | 232.4982 | 94.3964 | 0.7953 |

Shown is the residual covariance (${cov}_{e}$), the genetic covariance of the respective subset (${cov}_{set})$ and the corresponding 50K chip genetic covariance (${cov}_{50K}$) together with the standard errors (se) of these estimates. Also shown is the relative covariance explained by these three terms (${relcov}_{set}$*,* ${relcov}_{50K}$*,* ${relcov}_{e}$).

**Table S10 Absolute and relative covariances between milk yield and mastitis from model M4.**

| Subset | ${cov}_{set}$ | se ${cov}_{set}$ | ${relcov}_{set}$ | ${cov}_{50K}$ | se ${cov}_{50K}$ | ${relcov}_{50K}$ | ${cov}_{e}$ | se ${cov}_{e}$ | ${relcov}_{e}$ |
| --- | --- | --- | --- | --- | --- | --- | --- | --- | --- |
| young | -139.1501 | 73.4672 | -0.0686 | -1048.3258 | 124.6719 | -0.5165 | 842.2865 | 70.2337 | 0.4150 |
| untranslated regions | -392.8139 | 94.5153 | -0.1983 | -748.2447 | 123.5886 | -0.3776 | 840.3353 | 70.1918 | 0.4241 |
| splice sites | -112.4078 | 68.4442 | -0.0555 | -1068.2533 | 118.9472 | -0.5271 | 845.9887 | 70.1945 | 0.4174 |

Shown is the residual covariance (${cov}_{e}$), the genetic covariance of the respective subset (${cov}_{set})$ and the corresponding 50K chip genetic covariance (${cov}_{50K}$) together with the standard errors (se) of these estimates. Also shown is the relative covariance explained by these three terms (${relcov}_{set}$*,* ${relcov}_{50K}$*,* ${relcov}_{e}$).

**Table S11 Absolute and relative covariances between milk yield and digital phlegmon from model M4.**

| Subset | ${cov}_{set}$ | se ${cov}_{set}$ | ${relcov}_{set}$ | ${cov}_{50K}$ | se ${cov}_{50K}$ | ${relcov}_{50K}$ | ${cov}_{e}$ | se ${cov}_{e}$ | ${relcov}_{e}$ |
| --- | --- | --- | --- | --- | --- | --- | --- | --- | --- |
| young | -112.9121 | 74.0753 | -0.1067 | -471.6509 | 119.7471 | -0.4456 | 473.8766 | 75.7795 | 0.4477 |
| untranslated regions | -220.6202 | 90.9608 | -0.2155 | -333.1812 | 117.4580 | -0.3254 | 470.0914 | 75.7014 | 0.4591 |
| splice sites | -15.7224 | 67.5144 | -0.0151 | -558.2886 | 113.9604 | -0.5348 | 469.8363 | 75.7554 | 0.4501 |
| noncoding related | -2.0259 | 35.4156 | -0.0020 | -555.5416 | 104.0311 | -0.5454 | 461.0829 | 75.7404 | 0.4526 |

Shown is the residual covariance (${cov}_{e}$), the genetic covariance of the respective subset (${cov}_{set})$ and the corresponding 50K chip genetic covariance (${cov}_{50K}$) together with the standard errors (se) of these estimates. Also shown is the relative covariance explained by these three terms (${relcov}_{set}$*,* ${relcov}_{50K}$*,* ${relcov}_{e}$).

**Table S12 Absolute and relative covariances between milk yield and claw ulcers from model M4.**

| Subset | ${cov}_{set}$ | se ${cov}_{set}$ | ${relcov}_{set}$ | ${cov}_{50K}$ | se ${cov}_{50K}$ | ${relcov}_{50K}$ | ${cov}_{e}$ | se ${cov}_{e}$ | ${relcov}_{e}$ |
| --- | --- | --- | --- | --- | --- | --- | --- | --- | --- |
| young | 119.7623 | 90.0232 | 0.1130 | -419.1637 | 147.9612 | -0.3954 | 521.2411 | 84.3961 | 0.4917 |
| untranslated regions | -171.4326 | 110.9857 | -0.2087 | -120.6522 | 146.6801 | -0.1469 | 529.4065 | 84.3411 | 0.6444 |
| splice sites | 25.2893 | 81.0928 | 0.0289 | -325.2699 | 142.5859 | -0.3721 | 523.5581 | 84.3942 | 0.5990 |
| noncoding related | -16.1101 | 40.8594 | -0.0195 | -287.0419 | 129.7713 | -0.3471 | 523.8012 | 84.3403 | 0.6334 |

Shown is the residual covariance (${cov}_{e}$), the genetic covariance of the respective subset (${cov}_{set})$ and the corresponding 50K chip genetic covariance (${cov}_{50K}$) together with the standard errors (se) of these estimates. Also shown is the relative covariance explained by these three terms (${relcov}_{set}$*,* ${relcov}_{50K}$*,* ${relcov}_{e}$).

**Table S13 Results from the LD analysis of each subset and the 50K chip.**

| Subset | *decay intern* | *mean LD intern* | *decay extern* |  | *mean LD extern* | *distribution* | *mean MAF* |
| --- | --- | --- | --- | --- | --- | --- | --- |
| ChIPseq | 0.2338 | 0.1166 | 0.4071 |  | 0.2105 | 0.9925 | 0.1753 |
| conserved 100 | 0.2721 | 0.0867 | 0.4257 |  | 0.2050 | 0.9948 | 0.1640 |
| LD1 | 2.0580 | 0.0958 | 0.7641 |  | 0.1103 | 0.9945 | 0.1237 |
| LD2 | 0.9651 | 0.1497 | 0.6523 |  | 0.1706 | 0.9946 | 0.2022 |
| LD3 | 0.3564 | 0.2209 | 0.4549 |  | 0.2428 | 0.9945 | 0.2286 |
| LD4 | 0.0741 | 0.5071 | 0.3066 |  | 0.3827 | 0.9936 | 0.2434 |
| MAF2 | 0.5071 | 0.2654 | 0.4161 |  | 0.2100 | 0.9946 | 0.1208 |
| MAF3 | 0.5260 | 0.2816 | 0.4244 |  | 0.2483 | 0.9946 | 0.2366 |
| MAF4 | 0.5273 | 0.3910 | 0.4089 |  | 0.2718 | 0.9946 | 0.3927 |
| untranslated regions | 0.2063 | 0.1482 | 0.4239 |  | 0.2069 | 0.9940 | 0.1667 |
| VD1 | 0.3405 | 0.1560 | 0.4262 |  | 0.1804 | 0.9929 | 0.1869 |
| VD2 | 0.2686 | 0.1765 | 0.3659 |  | 0.2137 | 0.9936 | 0.1982 |
| VD3 | 0.2593 | 0.2127 | 0.3669 |  | 0.2438 | 0.9932 | 0.2056 |
| VD4 | 0.2704 | 0.2689 | 0.3927 |  | 0.2738 | 0.9921 | 0.2098 |
| aseQTL | 0.1987 | 0.2092 | 0.3940 |  | 0.2414 | 0.9872 | 0.2130 |
| coding related | 0.2345 | 0.1193 | 0.3992 |  | 0.2214 | 0.9909 | 0.1724 |
| eeQTL | 0.1774 | 0.2033 | 0.3737 |  | 0.2574 | 0.9911 | 0.2383 |
| geQTL | 0.1960 | 0.2806 | 0.4771 |  | 0.2227 | 0.9795 | 0.2444 |
| gene end | 0.2305 | 0.1392 | 0.4005 |  | 0.2301 | 0.9909 | 0.1785 |
| intron | 0.2763 | 0.1900 | 0.3957 |  | 0.2243 | 0.9896 | 0.2006 |
| mQTL | 0.4913 | 0.5087 | 0.6486 |  | 0.1700 | 0.9641 | 0.2701 |
| noncoding related | 0.1253 | 0.3886 | 0.3696 |  | 0.242 | 0.9967 | 0.1831 |
| sQTL | 0.1780 | 0.1818 | 0.3946 |  | 0.2442 | 0.9909 | 0.2223 |
| selection signatures | 0.3124 | 0.8548 | 0.3867 |  | 0.2118 | 0.9950 | 0.1185 |
| splice sites | 0.2389 | 0.1183 | 0.3831 |  | 0.2208 | 0.9980 | 0.1731 |
| young | 0.2374 | 0.2428 | 0.5461 |  | 0.2196 | 0.9894 | 0.2770 |

Shown are the decay (*decay intern*) and mean (*mean LD intern*) LD between the subset variants, the decay (*decay extern*) and mean (*mean LD extern*) LD between subset and adjacent sequence variants, and the mean minor allele frequency (*mean MAF*) of each subset. The parameter *distribution* indicates the distribution of the subset variants over the genome.
